# Genome-Wide Datasets of Chicories (*Cichorium intybus* L.) for Marker-Assisted Crop Breeding Applications: A Systematic Review and Meta-Analysis

**DOI:** 10.3390/ijms241411663

**Published:** 2023-07-19

**Authors:** Samela Draga, Giovanni Gabelli, Fabio Palumbo, Gianni Barcaccia

**Affiliations:** Department of Agronomy Food Natural Resources Animals Environment, Campus of Agripolis, University of Padova, 35020 Legnaro, Italy; samela.draga@phd.unipd.it (S.D.); giovanni.gabelli@unipd.it (G.G.)

**Keywords:** chicory, molecular markers, genetic maps, marker-assisted breeding, marker-assisted selection

## Abstract

*Cichorium intybus* L. is the most economically important species of its genus and among the most important of the Asteraceae family. In chicory, many linkage maps have been produced, several sets of mapped and unmapped markers have been developed, and dozens of genes linked to traits of agronomic interest have been investigated. This treasure trove of information, properly cataloged and organized, is of pivotal importance for the development of superior commercial products with valuable agronomic potential in terms of yield and quality, including reduced bitter taste and increased inulin production, as well as resistance or tolerance to pathogens and resilience to environmental stresses. For this reason, a systematic review was conducted based on the scientific literature published in chicory during 1980–2023. Based on the results obtained from the meta-analysis, we created two consensus maps capable of supporting marker-assisted breeding (MAB) and marker-assisted selection (MAS) programs. By taking advantage of the recently released genome of *C. intybus*, we built a 639 molecular marker-based consensus map collecting all the available mapped and unmapped SNP and SSR loci available for this species. In the following section, after summarizing and discussing all the genes investigated in chicory and related to traits of interest such as reproductive barriers, sesquiterpene lactone biosynthesis, inulin metabolism and stress response, we produced a second map encompassing 64 loci that could be useful for MAS purposes. With the advent of omics technologies, molecular data chaos (namely, the situation where the amount of molecular data is so complex and unmanageable that their use becomes challenging) is becoming far from a negligible issue. In this review, we have therefore tried to contribute by standardizing and organizing the molecular data produced thus far in chicory to facilitate the work of breeders.

## 1. Introduction

Chicories (2n = 2x = 18) are economically important dicot species belonging to the Asteraceae family. The Cichorium genus contains six main species, of which four are exclusively wild (*Cichorium bottae* Deflers., *Cichorium spinosum* L., *Cichorium calvum* Sch. Bip. ex Asch., and *Cichorium pumilum* Jacq.), one is exclusively cultivated (*Cichorium endivia* L.), and one contains both cultivated and wild individuals (*Cichorium intybus* L.) [1]. The main botanical variety in terms of economic impact is *Cichorium intybus* var. *foliosum*, widely appreciated for its leaves, which are eaten raw or cooked, and characterized by a distinctive bitter taste and crispiness. This variety includes ‘Witloof Chicory’ or ‘Belgian Endive’, commonly known in Europe for its typical etiolated buds named ‘chicon’, and Red Chicory, known as ‘Radicchio’, mostly distributed in northeastern Italy [2,3,4,5]. Apart from leaf chicory, mainly known for its nutritional and health-beneficial properties, an upsurge of interest has been observed in ‘industrial’ or ‘root’ chicory (*C. intybus* var. *sativum*), which is mostly used for inulin extraction and as a coffee substitute [6,7,8]. Inulin, as a carbohydrate reserve, accumulates during the first year’s growing season in taproot chicory and is used for food and nonfood applications [9,10].

The other economically relevant species from the same genus is *C. endivia*, whose curly and smooth leaves (var. *crispum* and var. *latifolium*, respectively) are consumed worldwide in fresh salads, with Spain, France, and Italy as major EU producers [9].

From a reproductive point of view, *Cichorium intybus* is a diploid plant species that is prevalently allogamous due to its efficient sporophytic self-incompatibility system [3,11,12]. Male sterility represents another efficient sexual barrier widely used in chicory to promote outcrossing and to facilitate the exploitation of heterosis through the production of F1 hybrids. In contrast, endive is an autogamous species with a rate of outcrossing of approximately 1% [13]. Chicory and endive, as closely related but distinct species, are completely interfertile and offer a vast genetic pool that, through cross-breeding schemes, might be exploited to obtain progeny with wide genetic diversity [3,4].

One of the main goals in chicory breeding programs is to achieve the best selections with valuable agronomic potential, such as yield, reduced bitter taste, increased inulin production, and resistance to both biotic and abiotic stressors. Molecular markers are fully addressed to assess genetic information on parental genotypes, heterozygosity evaluation and prediction, population uniformity and distinctiveness [13,14]. Moreover, these tools are employed not only in phylogenetic studies and genetic linkage map construction but also for the genetic traceability of the final commercial product [15,16]. As a result, the aim of generating a superior commercial product in accordance with market acceptance could be facilitated through the efficient implementation of marker-assisted breeding (MAB) and marker-assisted selection (MAS) programs [17,18,19].

In this study, after a systematic review of all the scientific literature produced for chicory in the last 40 years, the assembled genome of *Cichorium intybus* L. by Fan et al. was used to collect and physically map the genetic data available for this species [20]. The main advancements in chicory genetics and how they might be employed in breeding programs are the main topics of this study. To this aim, we developed two user-friendly genomic maps for breeding purposes. The first map contains single-nucleotide polymorphisms (SNPs) as well as simple-sequence repeats (SSRs), which might be helpful in MAB programs. The second map contains all the available gene sequences and marker-related genes, providing up-to-date information for MAS applications. The mapping of molecular markers and genes responsible for relevant agronomic traits has a significant impact on crop productivity and quality, and both maps are designed to act as a starting point for validating markers and genes of interest in chicory.

## 2. Methods

### 2.1. Literature Research

The systematic review and meta-analysis were performed according to the Preferred Reporting Items for Systematic Reviews and Meta-Analyses (PRISMA) guidelines. An overall bibliographic analysis for this work was conducted using Scopus and PubMed databases in January 2023. The search was confined to articles published between 1980 and 2023 and written in the English language. Since extensive data availability was expected, we split the research into two main parts representing MAB and MAS. A single list of articles was generated for MAB research, while 10 lists were produced for MAS, corresponding to the most relevant topics considered in chicory (namely, reproductive barriers, sesquiterpene lactone biosynthesis, hydroxycinnamates, inulin metabolism, stress response, blue-lilac color, flowering time, somatic embryogenesis, red discoloration and gene normalization). MAB-related research was conducted using key words as follows: (“*Cichorium intybus*” OR “chicory”) AND (“SSR” OR “microsatellite” OR “SNP” OR “SNV”). For the MAS part, the 10 most interesting topics were investigated using the specific terms reported in Table 1. After list collection, duplicates (i.e., overlapping results from both databases) were deleted and the eligibility assessment of the remaining articles was manually cured. We first excluded letters, conference papers, notes, articles without full-text availability, short reports and non-suitable articles based on the evaluation of abstracts and keywords. The remaining articles were deeply investigated and further filtered, by removing those either not fully consistent with the topic or lacking molecular data. In addition, we exploited the citations of the selected research papers, which led to an enrichment of the final reference list.

### 2.2. Data Collection and Maps Drawing

A comprehensive map for marker-assisted breeding purposes was built by using all the SSR- or SNP-containing sequences available in the scientific articles (identified according to the methods described in the previous section). The sequences, according to the indications provided by each article, were retrieved from GenBank and used as a query in the alignment against the *C. intybus* genome (JAK-NSD01 [20]). A BLASTn search was performed by setting the following parameters for SNPs-containing sequences: E-value, <1 × 10^−5^; percentage of identity, ≥95%; and minimum query coverage, 95%. For SSR primers, the following parameters were set: E-value, <1 × 10^−5^; percentage of identity, 100; and query coverage, 100%. The results were then filtered to retain only the five best hits for each query. Each query mapping with the same specificity and percentage of identity in more than one location and/or chromosome was discarded to avoid ambiguities. A consensus map was finally drawn using the ggplot2 (version 3.4.2) used in R environment version 4.2.0.

A second map for marker-assisted selection purposes was built by using all the genes available in the scientific articles (identified in accordance with the methods described in the previous section). The sequences were retrieved from GenBank and used as a query in the alignment against the *C. intybus* genome (JAK-NSD01 [20]). A BLASTn search was performed by setting the following parameters: E-value, <1 × 10^−5^; percentage of identity, ≥95; and minimum query coverage, 95%. When available, the closest SNP and SSR (both upstream and downstream) to each gene were selected too. A comprehensive map was finally drawn by using the ggplot2 R package and by plotting the genes along with the above-mentioned associated markers.

## 3. Results and Discussion

### 3.1. Screening Literature Results

**Figure 1:**
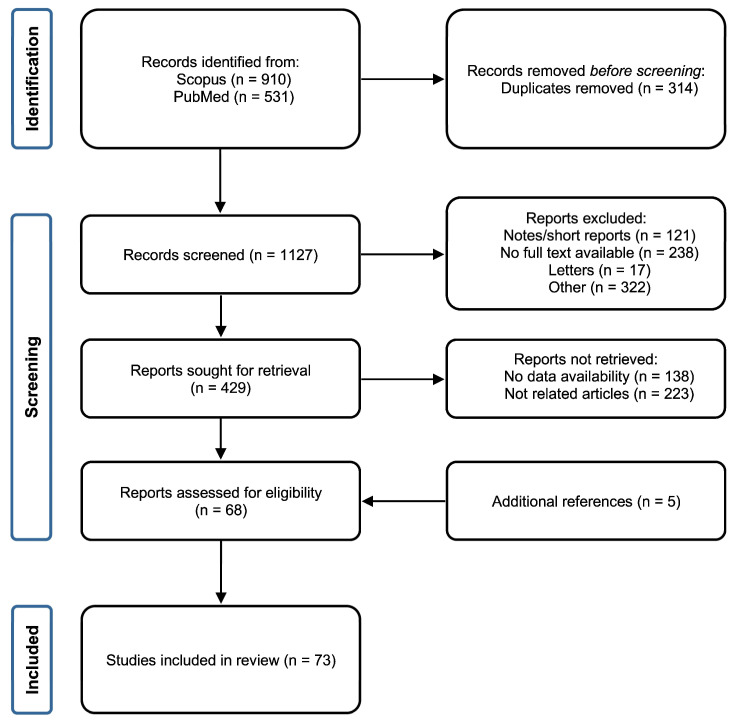
Flow chart of the screening and selection process followed for the inclusion of the studies in the systematic review (*n* denotes the number of studies resulting from each filtering step).

A preliminary survey of the literature led to the identification of 1441 records (910 from Scopus and 531 from PubMed). Briefly, 314 duplicate articles were removed, whereas 698 records including notes, articles without full-text availability, short surveys, and unrelated articles (based on abstract and keyword screening) were excluded. The remaining 429 results were analyzed based on their full content. From this filter, 361 were excluded because they did not match with the purpose of the study or data availability was limited or lacking. Five additional articles found in the references of the 68 remaining articles were added. Hence, the final reference list comprised 73 studies (Figure 1).

### 3.2. Toward a Genetic Genomic Map of Chicory Useful for Marker-Assisted Breeding

Linkage maps lay the groundwork for marker-assisted breeding. In chicory, the assembly of the first linkage map based on 371 markers (16 RAPDs, 72 SAMPLs and 283 AFLPs; 1201 cM) was reported in 1997 using an interspecific hybrid cross between *C. intybus* L. and an inbred line of *C. endivia* L. [21]. Similarly, Van Stallen et al. built a genetic linkage map based on an intraspecific cross between two inbred lines of witloof chicory (129 RAPD, 609.6 cM) [22]. A further RAPD-based genetic map was focused on the characterization of QTLs for the length and browning of pith [23]. Cassan et al. constructed a RAPD- and SSR-based genetic map (987 cM) to identify QTLs controlling physiological and agronomical traits under two levels of nitrogen fertilization during the vegetative phase of witloof chicory [24]. Unfortunately, all the above-mentioned maps, due to the type of markers (i.e., dominant markers), cannot be used for comparative studies. A turning point was the development by Cadalen et al. of a consensus genetic map from two industrial chicory progenies and one witloof chicory progeny, containing 472 SSR markers and covering 878 cM [25]. This study was further deepened by Gonthier et al. for the identification of markers associated with nuclear male sterility (NMS) and sporophytic self-incompatibility (SSI) loci [12]. Starting from the data produced in the two previous studies, Ghedina et al. developed an efficient multiplex assay for genotyping elite breeding stocks developed from old landraces of Radicchio of Chioggia [26]. This assay, composed of 27 SSR markers selected according to the polymorphism index and distribution, was further integrated with two additional SSR markers and successfully applied by Patella et al., 2019 [13]. Muys et al. produced a genetic map for industrial chicory that included 237 marker loci and spanned a total length of 1208 cM [27]. The map was built combining AFLPs, SSRs, SNPs, and 26 coding sequences. Finally, a high-density linkage map of leaf chicory was constructed using genotyping-by-sequencing (GBS) technology [28]. The map contained 727 SNP markers, covering a total length of 1413 cM. Most importantly, the map was pivotal for the identification of the putative locus responsible for male sterility. All the genetic maps produced in chicory are summarized in Table 2.

In addition to the aforementioned genetic maps, authors of several studies developed sets of unmapped markers, some of which had applications for a wide range of purposes. Thirty-one EST-SSRs with a high level of transferability potential between Cichorium species were proposed but never validated by Ince [29]. Raulier et al. developed a new set of 15 SSR marker loci to characterize the genetic diversity of the germplasm that originated in the current industrial chicory and to establish the relationships between and within chicory and endive species [1]. However, the sequences of this new set have never been made public by the authors. An additional set of 12 SSR markers was generated by Zavada et al. and used along with chloroplast DNA sequences to assess the temporal genetic changes and diversity in North America and in New England chicory populations [30,31].

Ideally, plant breeders draw on genetic maps and markers to mine information useful for MAB purposes [32]. However, as in the case of chicory, the availability of multiple linkage maps (each based on single and independent populations) and unmapped markers makes the interpretation and exploitation of the data very challenging. This challenge can be overcome through the production of consensus maps. Based on the procedure described in Section 2, we managed to position 639 markers within the physical map from Fan et al. [20], namely 579 SNPs and 60 SSRs derived from the studies of Cadalen et al., Ince, Muys et al., Zavada et al., Patella et al., and Palumbo et al. [13,25,27,28,29,30]. The newly developed consensus map is available in Figure S1.

**Figure 2:**
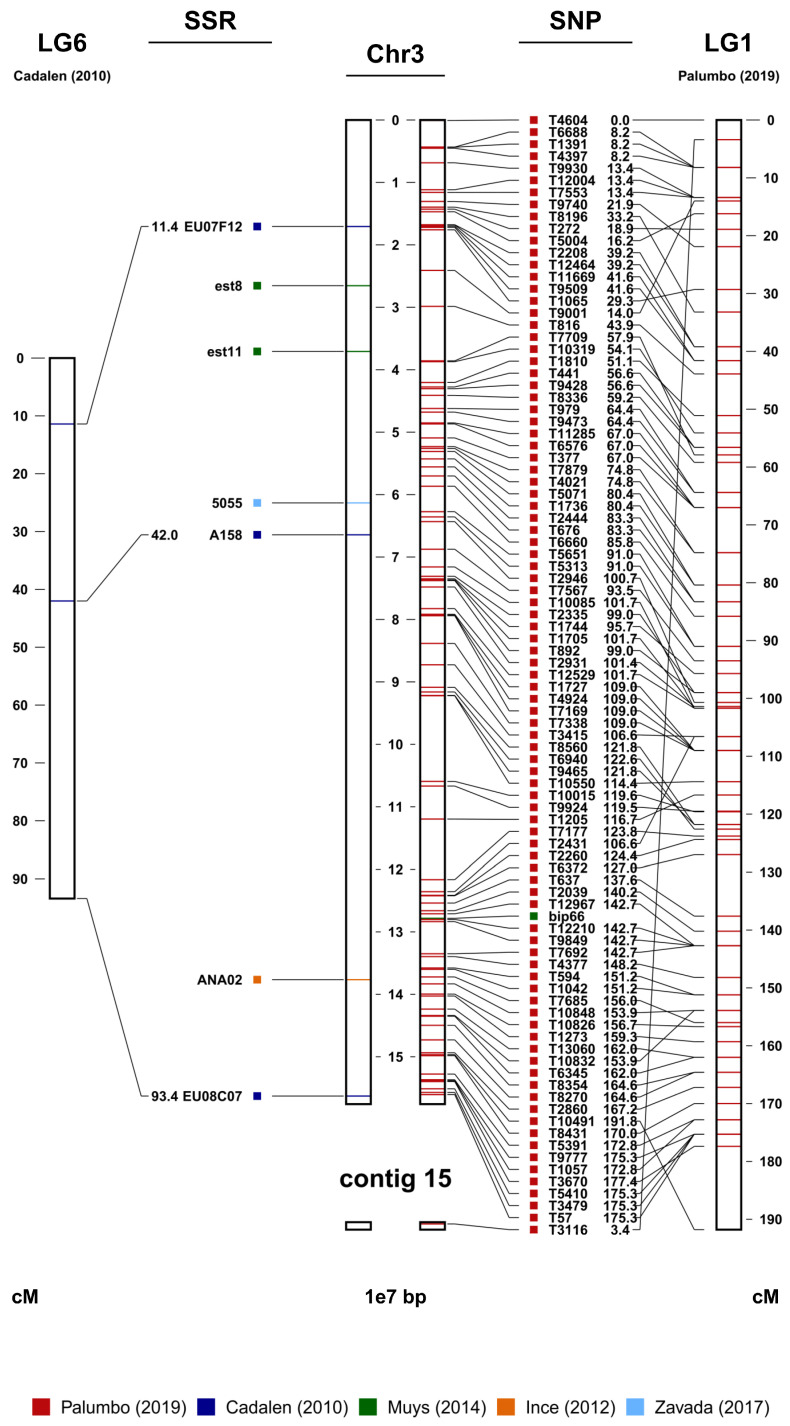
Chromosome 3 (in the center) from the marker-assisted breeding (MAB) map (the full MAB map is provided as Figure S1). On the left are the SSR sequences and their genetic distances in cM derived from LG6 of Cadalen et al. [25] and other unmapped SSRs from Ince et al. [29] and Zavada et al. [30]. On the right are all the available SNPs and LG1 of Palumbo et al. [28], with the respective genetic distances in cM, and the SNPs deriving from the work of Muys et al. [27]. Contig 15 was associated with chromosome 3 due to the mapping of the SNP marker on the right.

The observed discrepancy between the number of markers actually used for the consensus map and the total number of markers developed over the years essentially relies on two factors. The first, which is far from negligible, is that in many cases, the authors of the maps have not made (completely or partially) available the markers used. The second is that some of the available markers did not map. This last aspect is not surprising at all considering that almost all the markers were derived from extragenic and therefore less-conserved regions. This observation assumes particular relevance if we take into account that the different genetic maps and marker sets have been developed using interspecific hybrids (*C. endivia* × *C. intybus*) or different botanical varieties (*C. intybus* var. *sativum* and var. *foliosum*). The consensus map allowed us to anchor to specific chromosome positions those markers that until now lacked a position (i.e., all the unmapped markers), such as those from Ince and Zavada et al. [29,30]. Furthermore, the consensus map enabled us to establish the correspondences between the linkage groups of the genetic maps produced by Cadalen et al., Muys et al., and Palumbo et al., and the chromosomes assembled by Fan et al. (Table 2) [20,25,27,28]. An example is provided in Figure 2, where chromosome 3 (JAKNSD010000003.1) was found to correspond to LG6 of Cadalen et al. and LG1 of Palumbo et al. [20,25,28]. Within each linkage group/chromosome, most of the markers showed full collinearity. The cases in which collinearity was not observed could be the result of species/variety-specific rearrangements, errors in the construction of the genetic maps, or errors during genome assembly [32]. Finally, it should be noted that, thanks to the integration between the genetic maps and the physical map, it was possible to improve the latter by assigning some of the 199 unassembled contigs (JAKNSD010000010.1-JAKNSD010000208.1) to specific chromosomes. For example, contig 49 (JAKNSD010000049.1) in Figure S1 plausibly represents the terminal portion of chromosome 1 (JAKNSD010000001.1).

### 3.3. A Comprehensive Map for Marker-Assisted Selection Purposes

Linkage maps based on molecular markers also have the potential to bridge the gap between a given genotype and the resulting phenotype [33]. The basic principle of MAS is to identify a tight linkage between a marker and a gene controlling a trait of interest (e.g., disease resistance, plant cycle length, flowering time, or the reproductive system). This association can be used for practical purposes, including the preliminary screening of plant materials or to verify the introgression of a given gene. Thus, knowing the association with the gene of interest, traditional breeding methods, such as hybridization, backcrossing, self-pollination, and selection, are facilitated in the constitution of new varieties [34]. In this section, we first reviewed the scientific literature dealing with the identification of genes underlying ten topics of interest in *C. intybus*, as summarized in Table 3.

**Figure 3:**
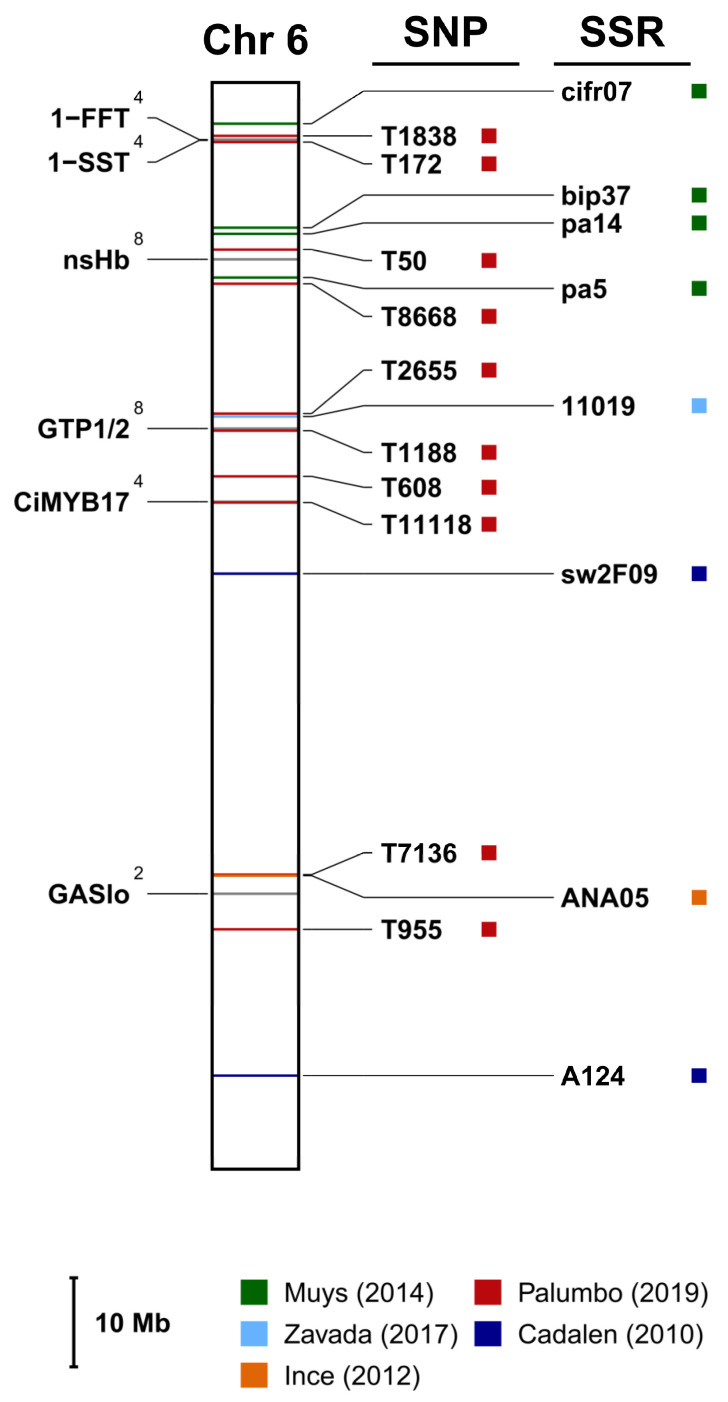
Chromosome 6 from the marker-assisted selection (MAS) map (the full MAS map is provided as Figure S2). On the left are the genes with their trait identity number (in the form of an apex: 2: sesquiterpene lactone biosynthesis (STL); 4: inulin metabolism; 8: somatic embryogenesis). On the right are reported the markers (SSRs or SNPs) closest to each gene and retrieved from Cadalen et al. [25], Muys et al. [27], Palumbo et al. [28], Ince et al. [29] and Zavada et al. [30]. Each gene is also described in detail in Table 3, whereas Table S1 reports their respective GenBank accession numbers.

According to the literature review described in Section 2, sixty-four sequences with a unique match with as many loci in the genome as possible were retrieved and are graphically represented in Figure S2. Moreover, when available, the closest SNP and SSR (both upstream and downstream) to each gene were mapped too. One of the chromosomes is shown as an example in Figure 3.

#### 3.3.1. Reproductive Barriers

The gene pool controlling the reproductive system of chicory is of great importance for hybrid development [12]. Some of the available genetic maps have been successfully used for the fine mapping of self-incompatibility and male sterility genes [12,25,28,35]. In root chicory, Gonthier et al. mapped the nuclear male sterility 1 (*NMS1*) locus on LG5 (namely, chromosome 7) [12]. Although the gene responsible for the lack of pollen has not yet been deciphered, the locus was finely confined to a region of 0.8 cM along with thirteen co-segregating AFLP markers. However, only two of them were transformed into SCAR markers named TGGC (1.03 cM) and ATGC (1.29 cM) and deposited in NCBI. In leaf chicory, the study of male sterility is at a more advanced stage. Initially, two SSR markers, M4.12 (acc. number, JF748831) and M4.11b (acc. number, KF880802), originally developed by Cadalen et al. and named EU02C09 and EU03H01, were found to be 5.8 cM and 12.1 cM away from the locus responsible for male sterility (*ms1*), respectively [25,28]. These two markers were located within LG4 from Cadalen et al., which corresponds to chromosome 9 [25]. Based on a SNP-based linkage map and synteny analysis of *Lactuca sativa*, it was finally possible to identify a four-nucleotide indel within the second exon of a *MYB103*-like gene responsible for an anticipated stop codon in male sterile mutants [28]. This gene, encoding a transcription factor involved in the callose dissolution of tetrads and exine development of microspores, was chosen as a candidate for male sterility.

In *C. intybus*, self-incompatibility was demonstrated more than 40 years ago [60]. From a molecular point of view, Gonthier et al. assigned the genetic determination of SSI to a single locus located in LG2 (corresponding to chromosome 5) [12]. Moreover, four AFLP-derived SCARs were assigned as TACG, GGAT, TTAA, and AACC due to their close association with the S-locus (the first two markers at 0.51 cM, the third at 0.39 cM and the fourth at 0.52 cM). Within this chromosomal region, Palumbo et al. recently identified *MDIS1 INTERACTING RECEPTOR LIKE KINASE 2* (*ciMIK2*) as the putative female determinant of SSI [36].

The availability of molecular genetic resources for reproductive barriers, such as self-incompatibility and male sterility, in chicory might have a great impact on the implementation of MAS programs to obtain highly heterozygous and phenotypically uniform progeny.

#### 3.3.2. Chicory, the Special Bitter-Taste Vegetable—STL Biosynthesis

**Figure 4:**
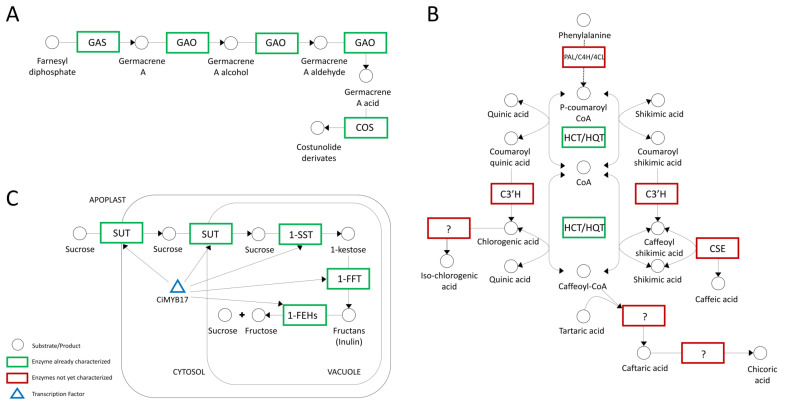
A schematic representation of three biosynthetic pathways of interest in chicory. Question marks indicate enzymes that have been characterized biochemically but are not supported by molecular data. (**A**) Sesquiterpene lactone (STL) biosynthetic pathway in chicory based on the studies of De Kraker et al. [64], Cankar et al. [39], Testone et al. [40], and Bogdanovic et al. [65]; (**B**) putative metabolic pathways involved in hydroxycinnamic acid (HCA) biosynthesis in plants and the two main enzymes HCT and HQT according to Legrand et al. [41]; (**C**) model of inulin metabolism, proposed by Van Laere and Van Den Ende [66] and Shoorideh et al. [67].

Sesquiterpene lactones (STLs) are secondary metabolites responsible for bitterness and have a significant active role in defense against pathogens [61]. From a nutritional standpoint, STLs also possess both beneficial (e.g., anticancer, and antileukemic) and allergenic properties [40,62]. Moreover, a recent study demonstrated that the biosynthesis pathway of sesquiterpene lactone (lactucin) is considered to provide antimalarial activity [63]. Three main enzymes, namely, germacrene A synthase (GAS), germacrene A oxidase (GAO) and costunolide synthase (COS), are involved in STL biosynthesis, as schematically represented in Figure 4A [64].

Due to the growing interest in these metabolites, several Asteraceae species have been investigated in this respect [38,39,40,68,69,70,71].

The sequences of three different GAS genes (*GASlo*, *GASsh* and *GAS1*) were retrieved and mapped on different chromosomes and they are represented in Figure S2 [37,40,65]. Initially, two isoenzymes referred to as long (GASlo) and short (GASsh) were isolated and characterized in chicory by Bouwmeester et al. [37]. The short form of the *GAS* gene was further confirmed via the study by Testone et al., named *GASsh2*, and published under a different accession number [40]. Indeed, *GASsh* and *GASsh2* mapped to the same position and are indicated as *GASsh/2* in Table S1 and Figure S2. *GASlo* and *GASsh* genes exhibited a relatively low degree of homology, although their enzymes catalyze the formation of the same product [37]. Based on the expression analysis, the transcripts of both genes were particularly abundant within root and seedling tissues, where the accumulation of bitter sesquiterpene lactones was indeed expected to be at the highest level [37,72]. However, it was reported that the *GASsh* (chromosome 2) gene was poorly expressed in leaves, while *GASlo* (chromosome 6) was expressed in both leaf and root tissues [65]. Testone et al. characterized a third gene, named the *GAS1* gene, that mapped to chromosome 5 [40]. From these results, the authors suggested the hypothesis of two distinct routes involved in STL synthesis and the involvement of different *GAS* genes [40].

Similarly, different *GAO* genes were identified via independent studies and were shown to convert germacrene A to its acid form [38,39,40]. According to Nguyen et al. and Testone et al., two of these sequences, generically named *GAO* (but deposited under different accession numbers), were mapped to the same position on chromosome 2 [38,40]. A third germacrene A oxidase gene, *CYP71AV8*, was mapped to chromosome 8 and was found to be involved in STL biosynthesis [39].

In the last step of STL biosynthesis, COS is crucial for the formation of costunolide derivates (Figure 4A). Testone et al., in addition to the *GAS* and *GAO* genes, also characterized a *COS* gene with the main idea of comprehending the regulation of bitter taste in chicories [40].

#### 3.3.3. Hydroxycinnamate Biosynthesis (HCA)

Hydroxycinnamates (HCAs) are secondary plant metabolites with phenylalanine as a precursor and are widely distributed in plants [41,73,74]. In chicory, interest is mostly focused on the biosynthesis of HCAs in the form of chlorogenic acid (CQA), isochlorogenic acid, (diCQA), caftaric acid (CTA), and chicoric acid (diCTA) (Figure 4B). These valuable molecules are responsible for many health benefits and are involved in plant protection against abiotic and biotic stresses [75,76,77]. Legrand et al. shed light on the genetic basis of HCA biosynthesis in *C. intybus*, isolating, cloning, and biochemically characterizing five full-length cDNA sequences encoding hydroxycinnamoyl-CoA: shikimate/quinate hydroxycinnamoyl transferases HCT1 and HCT2, and hydroxycinnamoyl-CoA/quinate hydroxycinnamoyl transferases HQT1, HQT2, and HQT3 [41]. Similarly, three *HQT*s and one *HCT* were discovered in artichoke, indicating the occurrence of several isoforms within these two gene families [41,78].

#### 3.3.4. Inulin Metabolism

Chicory root is one of the major natural sources of inulin, and this water-soluble storage polysaccharide belongs to a group of nondigestible carbohydrates called fructans [79]. Inulin can act as a substitute for fats and sugars and as a texture modifier, and it is becoming increasingly popular as a functional food ingredient [80,81]. The first year of the growing season is a crucial determinant for inulin accumulation in chicory [10]. Inulin metabolism, schematically simplified in Figure 4C, is mediated by fructan-active enzymes known as FAZYs, including sucrose 1-fructosyltransferase (1-SST) and fructan 1-fructosyltransferase (1-FFT), while inulin degradation is catalyzed by several isoforms of fructan 1-exohydrolases (1-FEH Is and 1-FEH IIs), which remove terminal fructose units [42,43,82].

A key factor in inulin accumulation is the allocation of sucrose as a substrate in the taproot [10,83]. In this first step, the main actors are sucrose uptake transporters (SUTs), which transfer sucrose from the source to the sink [10,84,85,86]. Wei et al. observed distinct expression profiles of three *SUT* genes whose sequences were submitted to GenBank [10]. We localized these sequences to chromosome 5 and 2. A transcription factor named CiMYB17 (chromosome 6) specifically activates the transcription of the *1-SST*, *1-FFT*, *1-FEH* and *SUT* genes (Figure 4C) [45]. Briefly, *1-SST* and *1-FFT* were both mapped to chromosome 6. Many independent studies have addressed instead the characterization of fructan 1-exohydrolases (1-FEHs). The cDNA of the fructan 1-exohydrolase I-coding gene (*1-FEH I*) of chicory (*Cichorium intybus* L.) was cloned, and its role was confirmed through its heterologous expression in potato [42]. Based on our mapping, *1-FEH I* is located on chromosome 9.

Two isoforms were initially thought to be responsible for 1-FEH II production: *1-FEH IIa* and *1-FEH IIb* [45]. These two sequences are located in LG4 of the Cadalen et al. map at a distance of 1.8 cM from each other and 3.8 cM away from the EU07G10 SSR marker [25]. The same markers resulted in chromosome 9 of the physical map of Fan et al. [20]. In parallel, Michiels et al. cloned and sequenced a cDNA derived from another putative 1-FEH IIa-coding gene (here renamed *1-FEH IIa2* and not to be confused with the original *1-FEH IIa* sequence mentioned above) and partially characterized its promoter region in a transient expression assay [44]. Similarly, a sequence analysis of the promoter region of another putative 1-FEH IIb-coding gene (here renamed *1-FEH IIb2* and not to be confused with the original *1-FEH IIb* sequence mentioned above) was conducted by Wei et al. [45]. To summarize, *1-FEH IIb* was mapped to chromosome 9, whereas the other three *1-FEH II* sequences (*1-FEH IIa*, *1-FEH IIa2*, and *1-FEHII b2*) retrieved from independent studies was mapped within contig 45 of Fan et al. (JAKNSD010000045.1 [20]) very closely to each other. However, the comparison between the genome assembly [20] and the GBS map by Palumbo et al. [28] supports the hypothesis that contig 45 is actually part of chromosome 9 (Figure S1). Thus, we can finally assume that the four *1-FEH II*-related sequences are all located on chromosome 9.

The specific role played by *1-FEH I*s and *1-FEH II*s in inulin degradation has not yet been fully elucidated. It was proposed that the induction of chicory *1-FEH I*s is mainly dependent on cold treatment, whereas that of *1-FEH II*s seems to be plausibly induced by both cold treatment and defoliation [10,42,43,45,66].

#### 3.3.5. Biotic and Abiotic Stresses

A significant number of studies have focused on biotic and abiotic stresses, demonstrating how crucial it is for breeding to understand the underlying mechanisms. In this section, we will discuss some of the actors involved in the stress crosstalk response, including nitrogen metabolism, protoporphyrinogen IX oxidase, dehydrins, dehydration-responsive element-binding protein (DREB), a novel vacuolar Na^+^/H^+^ exchanger gene and a corky root-related locus.

Nitrogen metabolism is one of the primary processes for plant growth, productivity, metabolism, and stress tolerance [87,88]. Nitrate reductase is the main actor in the nitrogen assimilatory pathway, catalyzing the two-electron reduction of nitrate to nitrite [89,90,91]. Using whole-mount in situ hybridization, Palms et al. demonstrated that young chicory plants show the spatial regulation of nitrate reductase gene (named *nia*) in their roots as a function of external nitrate concentration [46]. In this study, the full sequence of *nia* was isolated and characterized in chicory, and it was further suggested that the chicory genome contains a single *nia* gene (here mapped to chromosome 2) [46].

Protoporphyrinogen IX oxidase (protox, PPX1), a member of the protoporphyrinogen oxidase (PPO) family, catalyzes the conversion of protoporphyrinogen IX (protogen) into protoporphyrin IX (proto) [92,93]. PPO inhibitors prevent the formation of proto, causing protogen to accumulate in chloroplasts and leak into the cytosol, where it is nonenzymatically oxidized to proto [94,95]. Lipids and proteins are then oxidized, resulting in leaky membranes and the rapid disintegration of organelles and cells [94,96,97]. Adomat et al. isolated and sequenced the cDNA of plastidial *PPX1* from chicory [47]. The sequence was later mapped to LG1 in the map of Cadalen et al. [25] and on chromosome 4 in the physical map of Fan et al. [20].

Dehydrins are plant proteins known to be induced in response to environmental stresses (including drought, heat, freezing, metals/metalloids, or salinity), highlighting their potential role in biotechnological strategies to increase resistance in adverse environments [98,99,100,101,102]. Mingeot et al. identified two dehydrin cDNAs (*DHN1* and *DHN2*), both expressed in roots and leaves, with seasonal variations in transcript accumulation [48]. As dehydrins are involved in crosstalk processes, it would be interesting to further characterize and identify correlated genes expressed in response to abiotic stresses.

The *DREB1A* gene, belonging to the A-1 subtype of the DREB gene subfamily [103,104], has been identified as one of the most significant genes conferring tolerance in crops overcoming stressors [105,106]. To this aim, Lang et al. identified genes involved in abiotic stresses and reported their participation in ABA-independent stress signaling pathways in chicory [49,50]. The results revealed that two genes (*DREB1A* and *DREB2B*) were induced by low temperatures and that a novel vacuolar Na^+^/H^+^ exchanger gene (*CiNHX1*) was induced by salt stress [49,50].

Finally, Muys et al. identified the close association between the bip-41 SSR marker and *CAld5H* (coniferyl alcohol 5-hydroxylase) [27]. In lettuce, the same syntenic region encompassing both bip-41 and *CAld5H* also contains a recessive gene (*cor*) conferring resistance to *Rhizomonas suberifaciens*, the causal agent of corky root [27]. Further analyses are needed to investigate whether or not the same locus is also available in chicory.

#### 3.3.6. Lilac-Blue Color

Flavonoids are a large group of secondary metabolites ubiquitously present in plants [107,108]. They are mostly known for their role as pigments, with studies primarily focusing on the anthocyanin subgroup [8,109,110,111]. In addition, flavonoids are involved in protection against biotic and abiotic stress, in the regulation of developmental processes and in the integrity of the plant structure [8,110] Seitz et al. studied *F3′5′H* (flavonoid 3′,5′-hydroxylase) evolution from *F3′H* (flavonoid 3′-hydroxylase) in the Asteraceae family, probably triggered by an amino acid change at one specific position of the substrate recognition site [51]. The attainment of *F3′5′H* function allows the synthesis of delphinidin-based anthocyanins, which usually provide the basis for lilac to blue flower colors. The two sequences of *F3′H* and *F3′5′H*, obtained from this study, were retrieved and mapped nearby on chromosome 9.

#### 3.3.7. Flowering Time

The study of Périlleux et al. focused on the root chicory *FL1* gene, which belongs to the FLC/MAF clade of MADS box genes and behaves like the *AtFLC* gene, the repressor of flowering in Arabidopsis [52]. In this study, it was demonstrated that the *FL1* gene is downregulated in response to cold conditions and activated again at devernalizing temperatures. Eventually, the overexpression of *FL1* in Arabidopsis caused late flowering, but *FL1* repression was unstable when the postvernalization temperature was favorable for flowering and when the plants were devernalized. However, this instability of *FL1* repression may be related to the bienniality of root chicory as opposed to Arabidopsis’s annual lifecycle [52]. The *FL1* gene was mapped to chromosome 4.

#### 3.3.8. Somatic Embryogenesis (SE)

Somatic embryogenesis (SE) is an asexual propagation pathway requiring a transition of differentiated somatic cells toward embryogenic cells capable of producing embryos in a process resembling zygotic embryogenesis [112]. This mechanism holds great promise as a potential model in studies of early regulatory and morphogenetic events in plant embryogenesis [113]. Studies on gene expression were conducted in chicory comparing a SE-responsive genotype capable of undergoing complete cell reactivation, leading to somatic embryogenesis, with a non-SE-responsive genotype [114,115]. Genes possibly involved in somatic embryogenesis were first investigated through an extensive generation of expressed sequence tags (ESTs), but none of them turned out to be particularly promising [114,115].

Some interesting gene loci expressed during the early stages of somatic embryogenesis were studied in a *Cichorium* hybrid (*C. intybus* L., var. *sativum* × *C. endivia* L., var. *latifolium*) after the differential screening of a cDNA library in the leaf tissue [53]. Nonsymbiotic hemoglobin (*nsHb*) cDNA was isolated via Northern blot analysis. The gene was exclusively expressed under somatic embryogenesis-inducing conditions, excluding the correlation to stress caused by wounding or tissue culture conditions [53]. The sequence of this gene was further integrated into the map of Cadalen et al. (LG9 [25]) and on chromosome 6 (Figure S2).

Furthermore, β-1,3-glucanases, glutathione S-transferases and GTP binding proteins were investigated in independent studies as putative additional genes involved in SE mechanisms. Grimault et al. showed that during SE, callose β-1,3-glucanases were localized in the cell walls of embryogenic cells and embryos, suggesting a possible role in callose degradation [116]. After SE induction, Helleboid et al. isolated three different and possibly paralogous CG (callose glucanase) genes, all encoding β-1,3-glucanases [55]. The *CG2* and *CG3* cDNA sequences showed very high identity (98.5%), whereas they shared only 70% identity with *CG1*. In the map built and provided in Figure S2, these three genes were localized on chromosome 5, with *CG2* and *CG3* mapping in the same position (and referred to as *CG2/3*).

A cDNA encoding a glutathione S-transferase, *chi-GST1*, was isolated during the early stages of SE via a differential display in leaf tissues of chicory [54]. This led to the hypothesis that the transcript accumulation of *chi-GST1* was specific to the developing leaf of the SE cultivar, whereas no expression was observed in the leaf tissue of the non-SE-responsive cultivar [54]. Moreover, it was shown that *GST* genes were involved in a variety of processes, such as the detoxification of xenobiotic molecules, protection against the damaging effects of oxidative compounds resulting from cellular metabolism (such as lipid peroxidation), and the intracellular transport of nonsubstrate molecules [54,117,118].

Similarly, transcripts from leaf tissue explants of a SE-responsive chicory and a non-SE counterpart were compared to identify genes expressed during the early stages of SE [56]. By using the mRNA differential display method, two full-length GTP-binding protein cDNAs were expressed exclusively in the leaf tissue of the SE-responsive genotype. The two full-length *GTP1* and *GTP2* cDNAs differed by only 10 nucleotides, and the deduced proteins diverged by three amino acids. The two sequences were mapped to the same position on the physical map (Figure 3, chromosome 6, *GTP1/2*). *GTP1* and *GTP2* sequences might represent two alleles of the same gene, as suggested by the authors [56].

#### 3.3.9. Red Discoloration

Discoloration is a key postcutting trait that causes a loss of quality and consumer rejection [57,119,120,121,122]. In response to cutting, chicory gradually turns red, since tissue wounding induces the de novo synthesis of phenylalanine ammonia-lyase (PAL) and the activation of the phenylpropanoid pathway. The expression patterns of the genes encoding two phenylalanine ammonia-lyase (PAL) proteins (PAL1 and PAL2) were analyzed in postcut chicon tissues in response to heat treatment and controlled atmosphere storage [57]. PAL1 and PAL2 were strongly expressed in unheated cut tissues, whereas heat shock was found to reduce the level of *PAL1* transcripts in sliced endive, preventing discoloration. Transcript sequences of *PAL1* and *PAL2* were therefore included in the map reported in Figure S2, specifically in chromosomes 4 (*PAL1*) and 1 (*PAL2*) [57]. Comparable experiments in lettuce demonstrated that heat shock reduces the accumulation of *PAL* mRNAs and hence inhibits tissue browning in leaves [123].

#### 3.3.10. Gene Normalization

A critical step in the design of qRT—PCR experiments is the identification of reference genes. They are essential for data normalization and responsible for the accuracy of the data. Most importantly, the expression level of optimal reference genes should be comparable to that of the target genes, and expression should be stable under the chosen experimental conditions [58,59,124,125]. In chicory, Maroufi et al. identified seven candidate reference genes, namely, nicotinamide adenine dinucleotide dehydrogenase (*NADHD*), actin (*ACT*), β-tubulin (*TUB*), glyceraldehyde-3-phosphate-dehydrogenase (*GADPH*), histone H3 (*H3*), elongation factor 1-alpha (*EF*) and 18S rRNA (*rRNA*) [59]. Six of the seven sequences were available and are included in Figure S2. Similarly, Delporte et al., in a thorough investigation, analyzed 12 reference genes for data normalization, suitable for both cell cultures and seedlings [58]. Ten out of twelve were unequivocally matched with as many loci of the physical map of Fan et al. as possible [20].

## 4. Conclusions

Molecular advances in chicory represent priceless information that deserves to be properly collected, organized and stored for practical applications in breeding programs. Molecular marker technologies have been widely and successfully employed in many other horticultural crops and are considered extremely helpful for anticipating the selection process for plants with desirable traits. The reported results aimed to provide researchers with a simple mapping report that includes details on the genetic and physical locations of markers as well as additional results from other datasets that include genes and molecular markers. This led to the production of two easy-to-access consensus maps, one consisting of 639 molecular markers, useful for MAB applications, and a second reporting 64 sequences of genes or marker-related genes, useful for MAS purposes.

## Figures and Tables

**Table 1:** Keyword terms searched within the scientific literature databases. In addition to “*Cichorium intybus*” OR “chicory”, different keyword terms were applied to the selected databases according to the purpose of the research.

Purpose	Topic	Keywords Terms Searched
Development of a consensus map for marker-assisted breeding	Identification of SSR sequences	“SSR” OR “microsatellite”
	Identification of SNP sequences	“SNP” OR “SNV”
Development of a comprehensive map for marker-assisted selection	Reproductive barriers	“male-sterility” OR “self-incompatibility” OR “CMS” OR “NMS” OR “SSI” OR “S-locus”
	Sesquiterpene lactone biosynthesis	“sesquiterpene lactones” OR “STL” OR “lactucin”
	Hydroxycinnamates	“hydroxycinnamates” OR “HCA” OR “chlorogenic acid”
	Inulin metabolism	“inulin” OR “fructan”
	Stress response	“stressors” OR “stress” OR “biotic stress” OR “abiotic stress” OR “stress response” OR “stress tolerance”
	Blue-lilac color	“flavonoids” OR “anthocyanin” OR “flower color”
	Flowering time	“flowering” OR “flowering time” OR “flowering response”
	Somatic embryogenesis	“somatic embryogenesis” OR “SE”
	Red discoloration	“discoloration” OR “cuttings response”
	Gene normalization	“gene normalization” OR “reference genes” OR “data normalization”

**Table 2:** Genetic and physical maps developed over the years for Cichorium intybus. When available, for each map, we report the correspondence between each linkage group (LG) and the chromosome (Chr) of the consensus map (from Fan et al., 2022 [20]), the number and type of markers employed, the length of each map, and the taxonomy of the sample used for map construction.

References	De Simone et al. [21]	Van Stallen et al. [22]	Van Stallen et al. [23]	Cassan et al. [24]	Cadalen et al. [25]	Gonthier et al. [12]	Muys et al. [27]	Palumbo et al. [28]	Consensus Map (from Fan et al. [20])
LG ⟷ Chr	N.a.	N.a.	N.a.	N.a.	LG8	LG8	LG5	LG2	Chr1
					LG3	LG3	LG6	LG3	Chr2
					LG6	LG6	LG8	LG1	Chr3
					LG1	LG1	LG3	LG6	Chr4
					LG2	LG2	LG9	LG5	Chr5
					LG9	LG9	LG4	LG8	Chr6
					LG5	LG5	LG1	LG7	Chr7
					LG7	LG7	LG2	LG4	Chr8
					LG4	LG4	LG7	LG9	Chr9
Markers	16 RAPDs; 72 SAMPLs; 283 AFLPs	129 RAPDs	RAPDs	73 RAPDs; 9 SSRs	472 SSRs	SSRs; AFLPs	170 AFLPs; 28 SSRs; 27 EST-SNPs; 12 EST-SSRs	727 SNPs	579 SNPs; 60 SSRs
Length	1201 cM	609.6 cM	N.a.	987 cM	878 cM	N.a.	1208 cM	1413 cM	1280 Mb
Source	*C. intybus* (radicchio) × *C. endivia* (escarole)	*C. intybus* (witloof)	*C. intybus* (witloof)	*C. intybus* (witloof)	*C. intybus* (industrial); *C. intybus* (witloof)	*C. intybus* (industrial)	*C. intybus* (industrial);	*C. intybus* (radicchio)	*C. intybus* (Grassland Puna)

**Table 3:** The most relevant traits/features investigated in chicory, the responsible genes and/or the associated markers, and the methods used for their identification are reported. The superscript numbers (from 1 to 10) reported for each trait/topic were used to facilitate the correspondence with the genes shown in Figure 3 (for chromosome 6) and Figure S2 (for the entire chromosome set). The GenBank accession numbers of the genes/marker-related genes are reported in Table S1.

Traits/Topic	Gene or Marker Locus	Methods	Citation
Reproductive barriers ^1^	*NMS-related* (*NMS1*, *NMS2*)	Genetic mapping (AFLP-based SCARs)	[12]
	*ms1* (*MYB103-like*)	Genetic mapping (SSR; SNP)	[25,28,35]
	*S-related* (*S1-S4*), *MIK2*	Genetic mapping (AFLP-based SCARs)	[12,36]
Sesquiterpene lactone biosynthesis (STL) ^2^	*GASlo*, *GASsh*	cDNA library construction; expression analysis in *E. coli*	[37]
	*GAO*	Analyses of the metabolites from transgenic yeast; immunoblot analysis; in vitro enzyme assay; GC—MS enzyme assay	[38]
	*CYP71AV8*	Isolation; cloning; coexpression in yeasts; GC—MS analysis	[39]
	*GASsh2*, *GAS1*, *GAO*, *COS*	RNA sequencing, transcriptome assembly, functional annotation, gene expression analyses, qPCR	[40]
Hydroxycinnamates (HCAs) ^3^	*HCT1*, *HCT2*, *HQT1*, *HQT2*, *HQT3*	In vitro assays of recombinant proteins in *E. coli*, transient expression in *N. benthamiana*, SDS—PAGE and immunoblot analysis	[41]
Inulin metabolism ^4^	*1-FEH I*	Cloning, MALDI-TOF and Q-TOF analyses, and expression in transgenic potato tubers	[42]
	*1-FEH IIa*, *1-FEH IIb*	Sequencing, Q-TOF analyses, RNA isolation, RT—PCR, and subcloning	[43]
	*1-FEH IIa2*	Northern blot hybridization, Transient expression analysis, Promoter analysis	[44]
	*1- FEH IIb2*, *CiMYB17*, *SUT1*, *SUT2*, *SUT3*,*1-SST*, *1-FFT*	RNAseq, yeast one-hybrid assay, transfection experiments, transient expression in grapevine (*Vitis vinifera*), and qPCR	[10,45]
Stress response ^5^	*nia* gene	Northern blot analysis, ln-situ hybridization, cloning, and sequencing	[46]
	*PPX1*	Ion exchange chromatography, construction of a cDNA library, cloning, Expression analysis in *E. coli*, and ProTox assay	[47]
	*CAld5H* (*bip41*)	Genetic mapping (SNP)	[27]
	*DHN1*, *DHN2*	Southern blot analysis, Northern blot analysis, cloning, and transcription promoter analysis	[48]
	*CiNHX1*	Cloning of *CiDREB1*, transformation in *E. coli*, qPCR, and subcellular localization	[49]
	*DREB1A*, *DREB1B*	Subcloning, sequencing, qPCR, protein localization, and functional expression in a yeast mutant	[50]
Blue-lilac color ^6^	*F3’H*, *F3’5’H*	Extraction of anthocyanins, cloning of F3’H and F3 5 H cDNAs, and expression analysis in yeast	[51]
Flowering time ^7^	*FL1 gene*	Cloning, qPCR, construction of transgenics, and transformation in Arabidopsis via floral dip method	[52]
Somatic embryogenesis ^8^	*nsHb*	Construction of cDNA library in a phage lambda and integration in *E. coli*, differential screening of the cDNA library, and Northern blot analysis	[53]
	*chi-GST1*	RT—PCR, Northern blot analysis, and expression of the protein in *E. coli*,	[54]
	*CG1*	qPCR, RACE PCR, in vivo expression of cDNAs in *Escherichia coli*, Southern blot analysis, and Northern blot analysis	[55]
	*GTP1/2*	Cloning, library construction and screening, Northern blot analysis, (RACE) PCR, RT—PCR, and expression analysis in BL21 bacterial cells	[56]
Red discoloration ^9^	*PAL1*, *PAL2*	Color analysis, RNA extraction, cDNA synthesis, and qPCR	[57]
Gene normalization ^10^	*βTUB*, *UBQ10*, *SAND*, *Clath*, *TIP41*, *PP2AA3*, *CYP5*, *ACT2*, *PROF*, *ACT7*	Determination of reference gene expression stability using geNorm, NormFinder and BestKeeper	[58]
	*ACT*, *EF-1αM*, *NADHD*, *His-H3*, *rRNA*, *TUB*	Determination of reference gene expression stability using geNorm, NormFinder and BestKeeper	[59]

## Data Availability

Not applicable.

## Supplementary Materials

The following supporting information can be downloaded at: https://www.mdpi.com/article/10.3390/ijms241411663/s1.
