# Supplementary material for: Genome-Wide Datasets of Chicories (Cichorium intybus L.) for Marker-Assisted Crop Breeding Applications: A Systematic Review and Meta-Analysis
Source: Int J Mol Sci. 2023 Jul 19;24(14):11663. doi: 10.3390/ijms241411663 (PMC10380310; doi:10.3390/ijms241411663)
Supplement: Supplementary file 1 [file ijms-24-11663-s001.zip › Figure S1.pdf]

Figure S1: Marker Assisted Breeding (MAB) map representing the position of SSR and SNP markers, developed by various authors on the chicory genome assembly JAKNSD01 (in the center). Specifically, on the left are the SSR sequences (by the authors: Cadalen et al. [25], Ince et al. [29], Patella et al., [13], and Zavada et al., [30]), while on the right are all the SNPs markers retrieved from the map built by Palumbo et al [28] and Muys et al., [27]. For the SSR marker from Cadalen et al. [25], and the SNP markers from Palumbo et al. [28], their position on the corresponding linkage group is also graphically indicated, distance in cM (on the flanks of the figure). The contigs are assigned to their respective chromosomes due to the mapping of the markers.

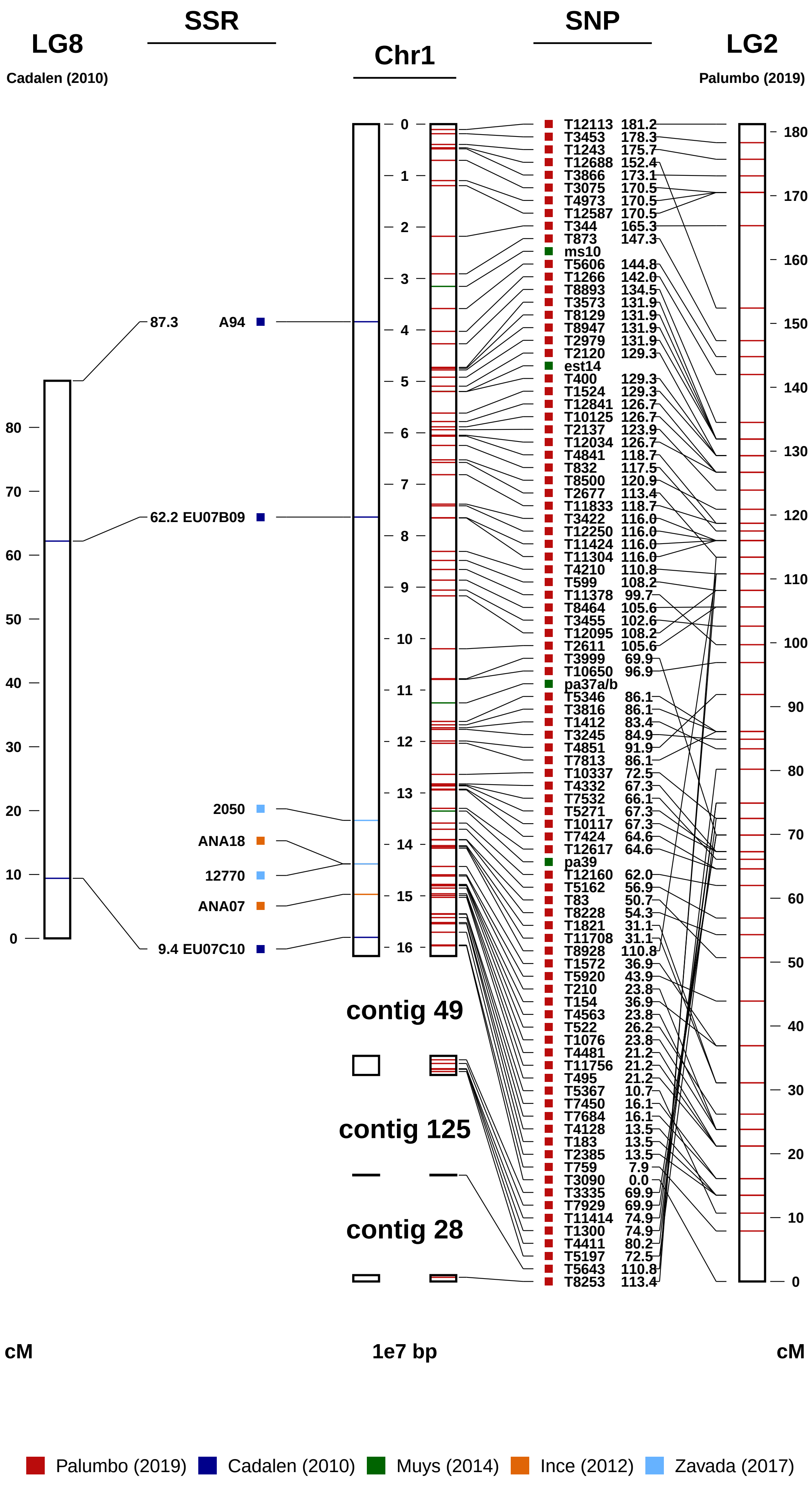

SSR
SNP
LG8
Cadalen (2010)
Chr1
LG2
Palumbo (2019)
87.3 A94
62.2 EU07B09
2050
ANA18
12770
ANA07
9.4 EU07C10
80
70
60
50
40
30
20
10
0
0
1
2
3
4
5
6
7
8
9
10
11
12
13
14
15
16
contig 49
contig 125
contig 28
T12113 181.2
T3453 178.3
T1243 175.7
T12688 152.4
T3866 173.1
T3075 170.5
T4973 170.5
T12587 170.5
T344 165.3
T873 147.3
ms10
T5606 144.8
T1266 142.0
T8893 134.5
T3573 131.9
T8129 131.9
T8947 131.9
T2979 131.9
T2120 129.3
est14
T400 129.3
T1524 129.3
T12841 126.7
T10125 126.7
T2137 123.9
T12034 126.7
T4841 118.7
T832 117.5
T8500 120.9
T2677 113.4
T11833 118.7
T3422 116.0
T12250 116.0
T11424 116.0
T11304 116.0
T4210 110.8
T599 108.2
T11378 99.7
T8464 105.6
T3455 102.6
T12095 108.2
T2611 105.6
T3999 69.9
T10650 96.9
pa37a/b
T5346 86.1
T3816 86.1
T1412 83.4
T3245 84.9
T4851 91.9
T7813 86.1
T10337 72.5
T4332 67.3
T7532 66.1
T5271 67.3
T10117 67.3
T7424 64.6
T12617 64.6
pa39
T12160 62.0
T5162 56.9
T83 50.7
T8228 54.3
T1821 31.1
T11708 31.1
T8928 110.8
T1572 36.9
T5920 43.9
T210 23.8
T154 36.9
T4563 23.8
T522 26.2
T1076 23.8
T4481 21.2
T11756 21.2
T495 21.2
T5367 10.7
T7450 16.1
T7684 16.1
T4128 13.5
T183 13.5
T2385 13.5
T759 7.9
T3090 0.0
T3335 69.9
T7929 69.9
T11414 74.9
T1300 74.9
T4411 80.2
T5197 72.5
T5643 110.8
T8253 113.4
180
170
160
150
140
130
120
110
100
90
80
70
60
50
40
30
20
10
0
cM
1e7 bp
cM
Palumbo (2019)
Cadalen (2010)
Muys (2014)
Ince (2012)
Zavada (2017)

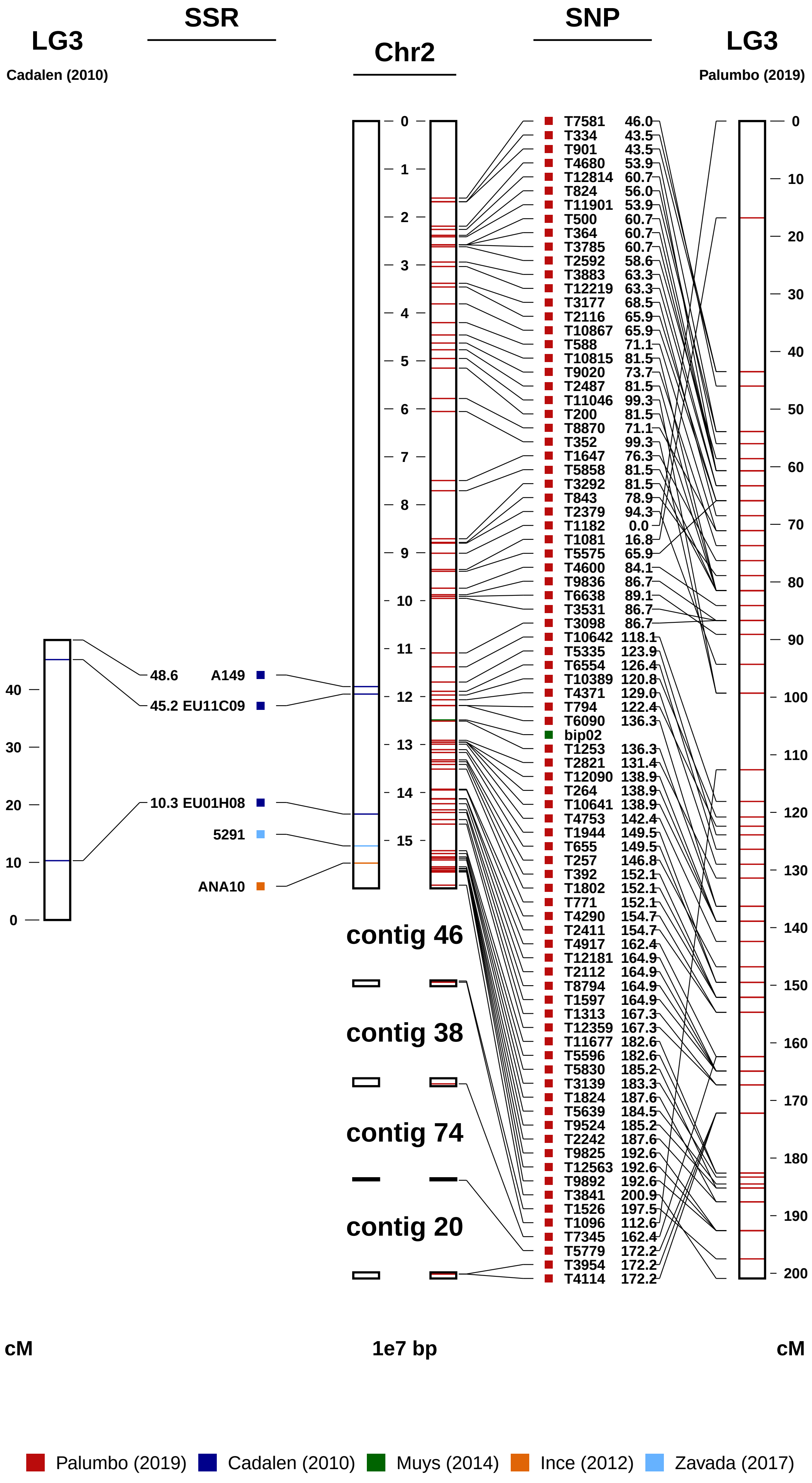
LG3
Cadalen (2010)
SSR
Chr2
SNP
LG3
Palumbo (2019)
48.6 A149
45.2 EU11C09
10.3 EU01H08
5291
ANA10
contig 46
contig 38
contig 74
contig 20
T7581 46.0
T334 43.5
T901 43.5
T4680 53.9
T12814 60.7
T824 56.0
T11901 53.9
T500 60.7
T364 60.7
T3785 60.7
T2592 58.6
T3883 63.3
T12219 63.3
T3177 68.5
T2116 65.9
T10867 65.9
T588 71.1
T10815 81.5
T9020 73.7
T2487 81.5
T11046 99.3
T200 81.5
T8870 71.1
T352 99.3
T1647 76.3
T5858 81.5
T3292 81.5
T843 78.9
T2379 94.3
T1182 0.0
T1081 16.8
T5575 65.9
T4600 84.1
T9836 86.7
T6638 89.1
T3531 86.7
T3098 86.7
T10642 118.1
T5335 123.9
T6554 126.4
T10389 120.8
T4371 129.0
T794 122.4
T6090 136.3
bip02
T1253 136.3
T2821 131.4
T12090 138.9
T264 138.9
T10641 138.9
T4753 142.4
T1944 149.5
T655 149.5
T257 146.8
T392 152.1
T1802 152.1
T771 152.1
T4290 154.7
T2411 154.7
T4917 162.4
T12181 164.9
T2112 164.9
T8794 164.9
T1597 164.9
T1313 167.3
T12359 167.3
T11677 182.6
T5596 182.6
T5830 185.2
T3139 183.3
T1824 187.6
T5639 184.5
T9524 185.2
T2242 187.6
T9825 192.6
T12563 192.6
T9892 192.6
T3841 200.9
T1526 197.5
T1096 112.6
T7345 162.4
T5779 172.2
T3954 172.2
T4114 172.2
cM
1e7 bp
cM
Palumbo (2019)
Cadalen (2010)
Muys (2014)
Ince (2012)
Zavada (2017)

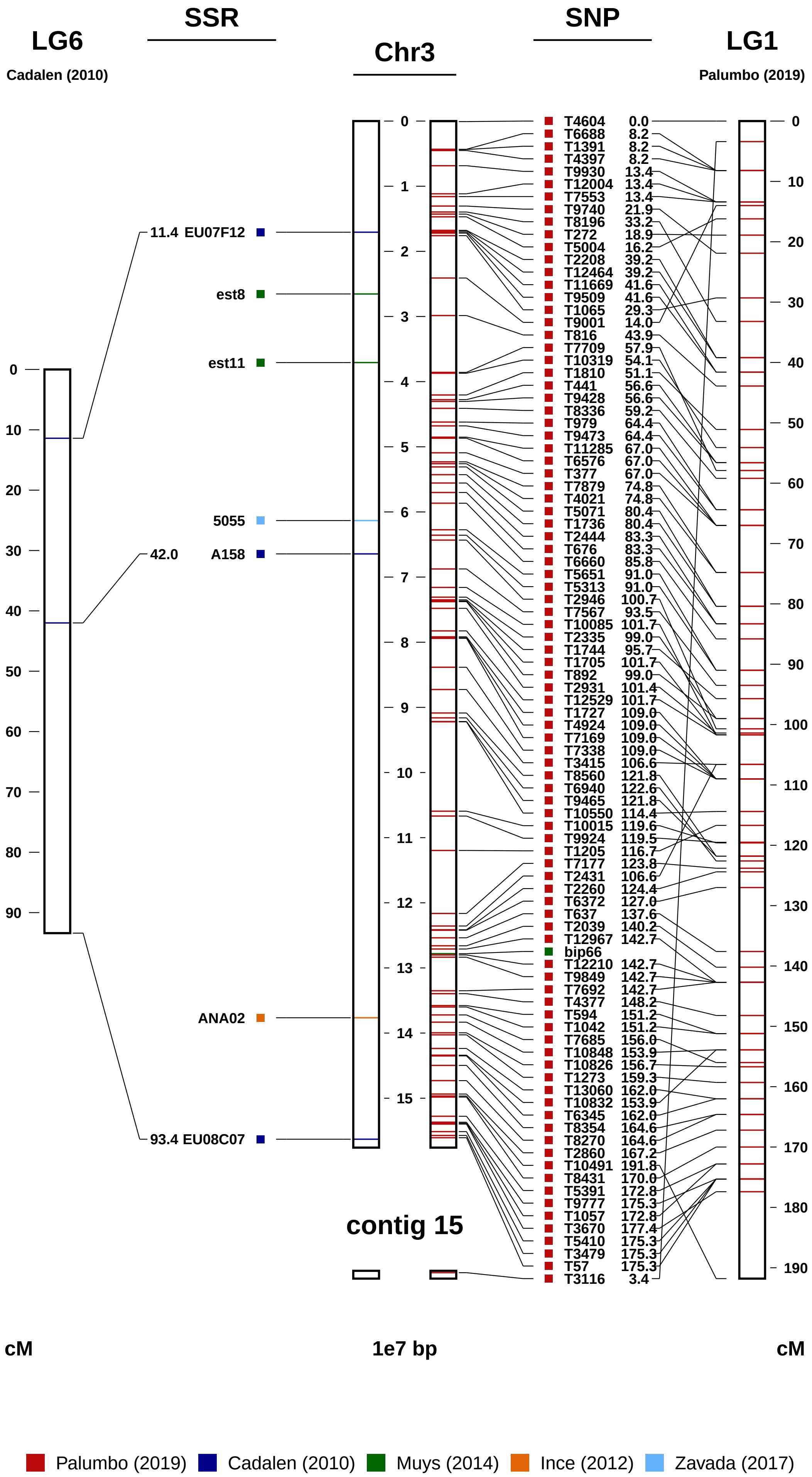

LG6
Cadalen (2010)
SSR
Chr3
SNP
LG1
Palumbo (2019)
11.4 EU07F12
est8
est11
5055
42.0 A158
ANA02
93.4 EU08C07
contig 15
T4604 0.0
T6688 8.2
T1391 8.2
T4397 8.2
T9930 13.4
T12004 13.4
T7553 13.4
T9740 21.9
T8196 33.2
T272 18.9
T5004 16.2
T2208 39.2
T12464 39.2
T11669 41.6
T9509 41.6
T1065 29.3
T9001 14.0
T816 43.9
T7709 57.9
T10319 54.1
T1810 51.1
T441 56.6
T9428 56.6
T8336 59.2
T979 64.4
T9473 64.4
T11285 67.0
T6576 67.0
T377 67.0
T7879 74.8
T4021 74.8
T5071 80.4
T1736 80.4
T2444 83.3
T676 83.3
T6660 85.8
T5651 91.0
T5313 91.0
T2946 100.7
T7567 93.5
T10085 101.7
T2335 99.0
T1744 95.7
T1705 101.7
T892 99.0
T2931 101.4
T12529 101.7
T1727 109.0
T4924 109.0
T7169 109.0
T7338 109.0
T3415 106.6
T8560 121.8
T6940 122.6
T9465 121.8
T10550 114.4
T10015 119.6
T9924 119.5
T1205 116.7
T7177 123.8
T2431 106.6
T2260 124.4
T6372 127.0
T637 137.6
T2039 140.2
T12967 142.7
bip66
T12210 142.7
T9849 142.7
T7692 142.7
T4377 148.2
T594 151.2
T1042 151.2
T7685 156.0
T10848 153.9
T10826 156.7
T1273 159.3
T13060 162.0
T10832 153.9
T6345 162.0
T8354 164.6
T8270 164.6
T2860 167.2
T10491 191.8
T8431 170.0
T5391 172.8
T9777 175.3
T1057 172.8
T3670 177.4
T5410 175.3
T3479 175.3
T57 175.3
T3116 3.4
cM
1e7 bp
cM
Palumbo (2019)
Cadalen (2010)
Muys (2014)
Ince (2012)
Zavada (2017)

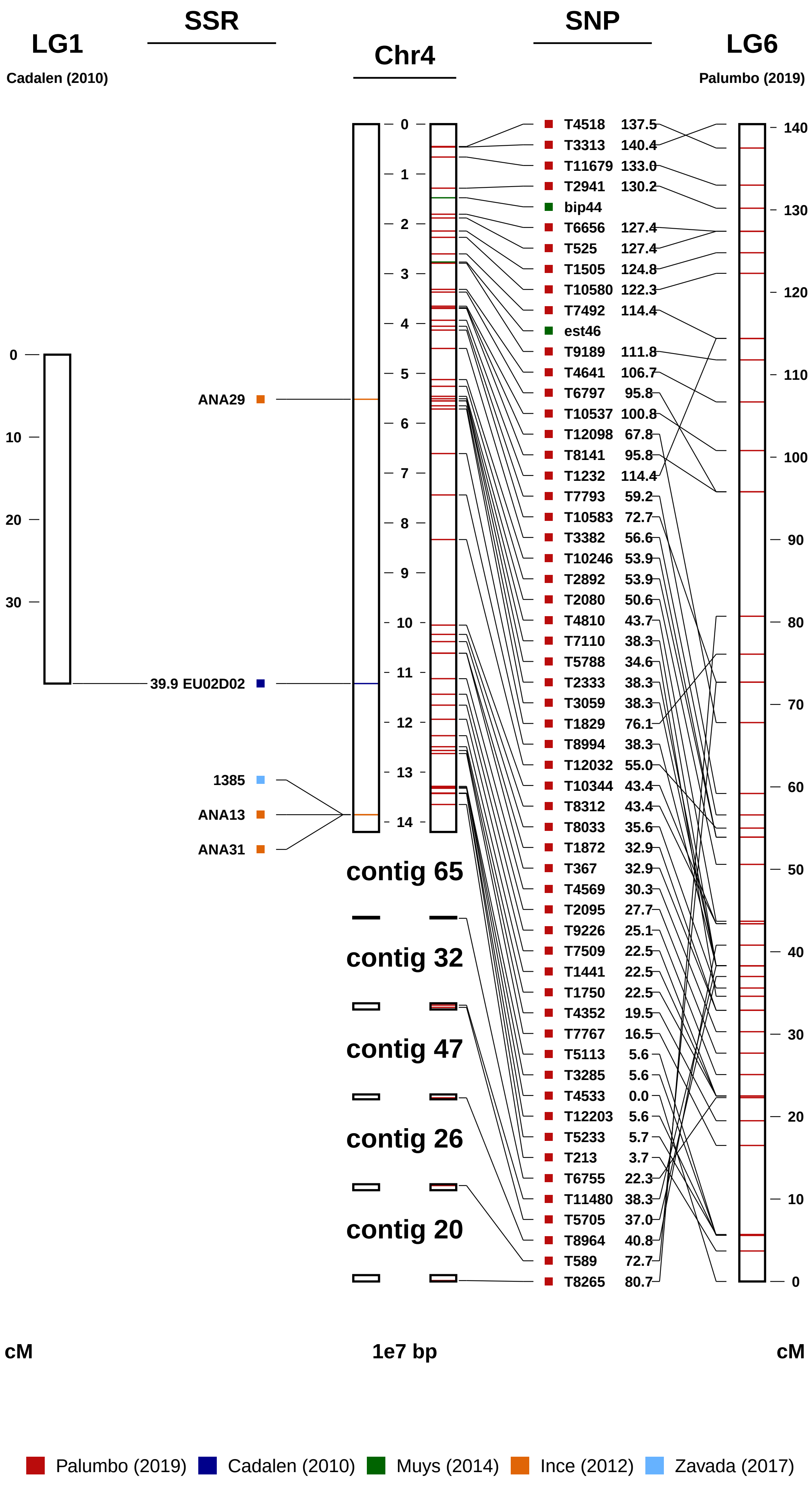

LG1
Cadalen (2010)
SSR
Chr4
SNP
LG6
Palumbo (2019)
0
10
20
30
ANA29
39.9 EU02D02
1385
ANA13
ANA31
0 1 2 3 4 5 6 7 8 9 10 11 12 13 14
contig 65
contig 32
contig 47
contig 26
contig 20
T4518 137.5
T3313 140.4
T11679 133.0
T2941 130.2
bip44
T6656 127.4
T525 127.4
T1505 124.8
T10580 122.3
T7492 114.4
est46
T9189 111.8
T4641 106.7
T6797 95.8
T10537 100.8
T12098 67.8
T8141 95.8
T1232 114.4
T7793 59.2
T10583 72.7
T3382 56.6
T10246 53.9
T2892 53.9
T2080 50.6
T4810 43.7
T7110 38.3
T5788 34.6
T2333 38.3
T3059 38.3
T1829 76.1
T8994 38.3
T12032 55.0
T10344 43.4
T8312 43.4
T8033 35.6
T1872 32.9
T367 32.9
T4569 30.3
T2095 27.7
T9226 25.1
T7509 22.5
T1441 22.5
T1750 22.5
T4352 19.5
T7767 16.5
T5113 5.6
T3285 5.6
T4533 0.0
T12203 5.6
T5233 5.7
T213 3.7
T6755 22.3
T11480 38.3
T5705 37.0
T8964 40.8
T589 72.7
T8265 80.7
140 130 120 110 100 90 80 70 60 50 40 30 20 10 0
cM
1e7 bp
cM
Palumbo (2019)
Cadalen (2010)
Muys (2014)
Ince (2012)
Zavada (2017)

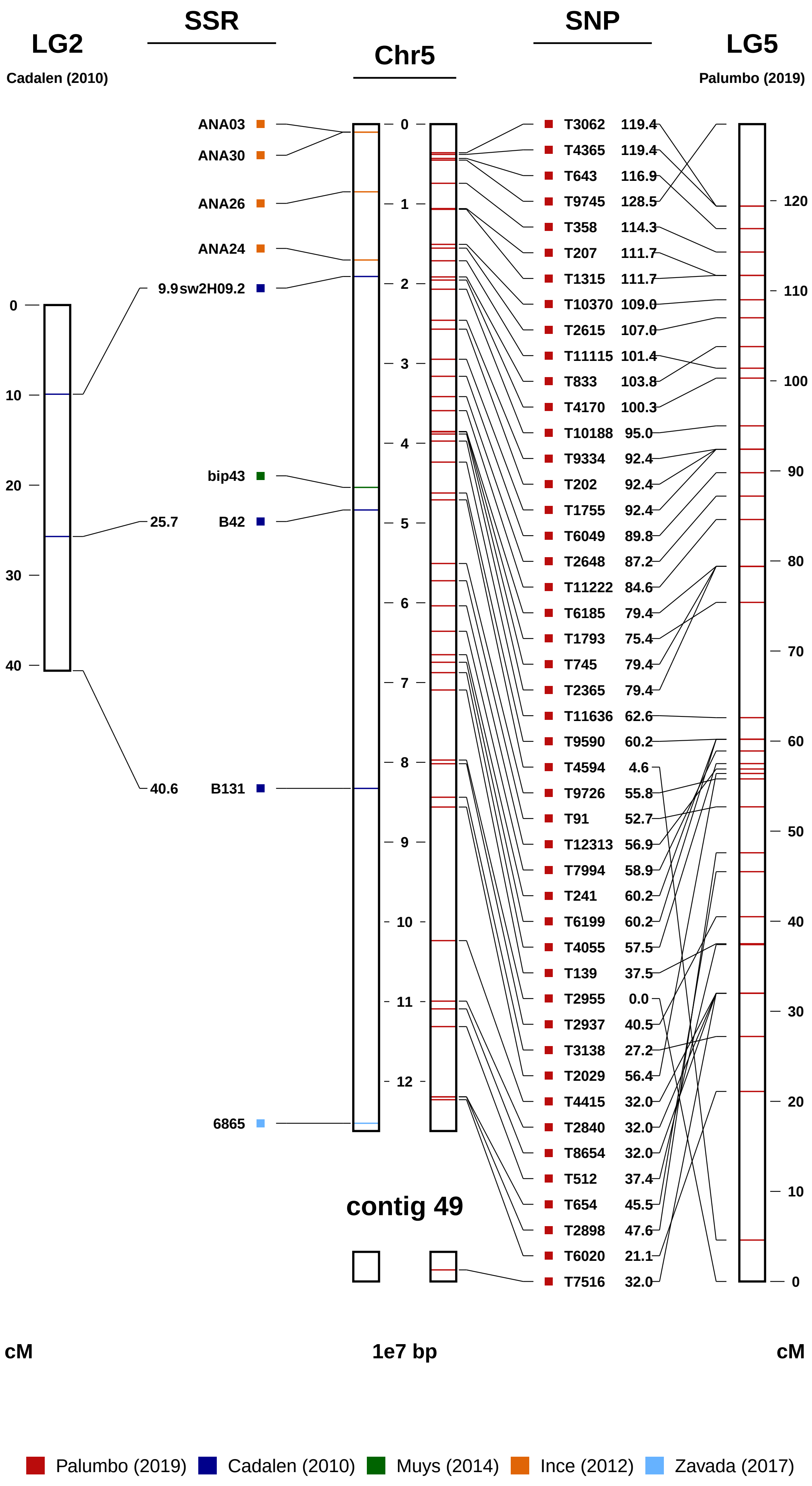

LG2
Cadalen (2010)
SSR
Chr5
SNP
LG5
Palumbo (2019)
ANA03
ANA30
ANA26
ANA24
9.9 sw2H09.2
bip43
25.7 B42
40.6 B131
6865
T3062 119.4
T4365 119.4
T643 116.9
T9745 128.5
T358 114.3
T207 111.7
T1315 111.7
T10370 109.0
T2615 107.0
T11115 101.4
T833 103.8
T4170 100.3
T10188 95.0
T9334 92.4
T202 92.4
T1755 92.4
T6049 89.8
T2648 87.2
T11222 84.6
T6185 79.4
T1793 75.4
T745 79.4
T2365 79.4
T11636 62.6
T9590 60.2
T4594 4.6
T9726 55.8
T91 52.7
T12313 56.9
T7994 58.9
T241 60.2
T6199 60.2
T4055 57.5
T139 37.5
T2955 0.0
T2937 40.5
T3138 27.2
T2029 56.4
T4415 32.0
T2840 32.0
T8654 32.0
T512 37.4
T654 45.5
T2898 47.6
T6020 21.1
T7516 32.0
contig 49
cM
1e7 bp
cM
Palumbo (2019)
Cadalen (2010)
Muys (2014)
Ince (2012)
Zavada (2017)

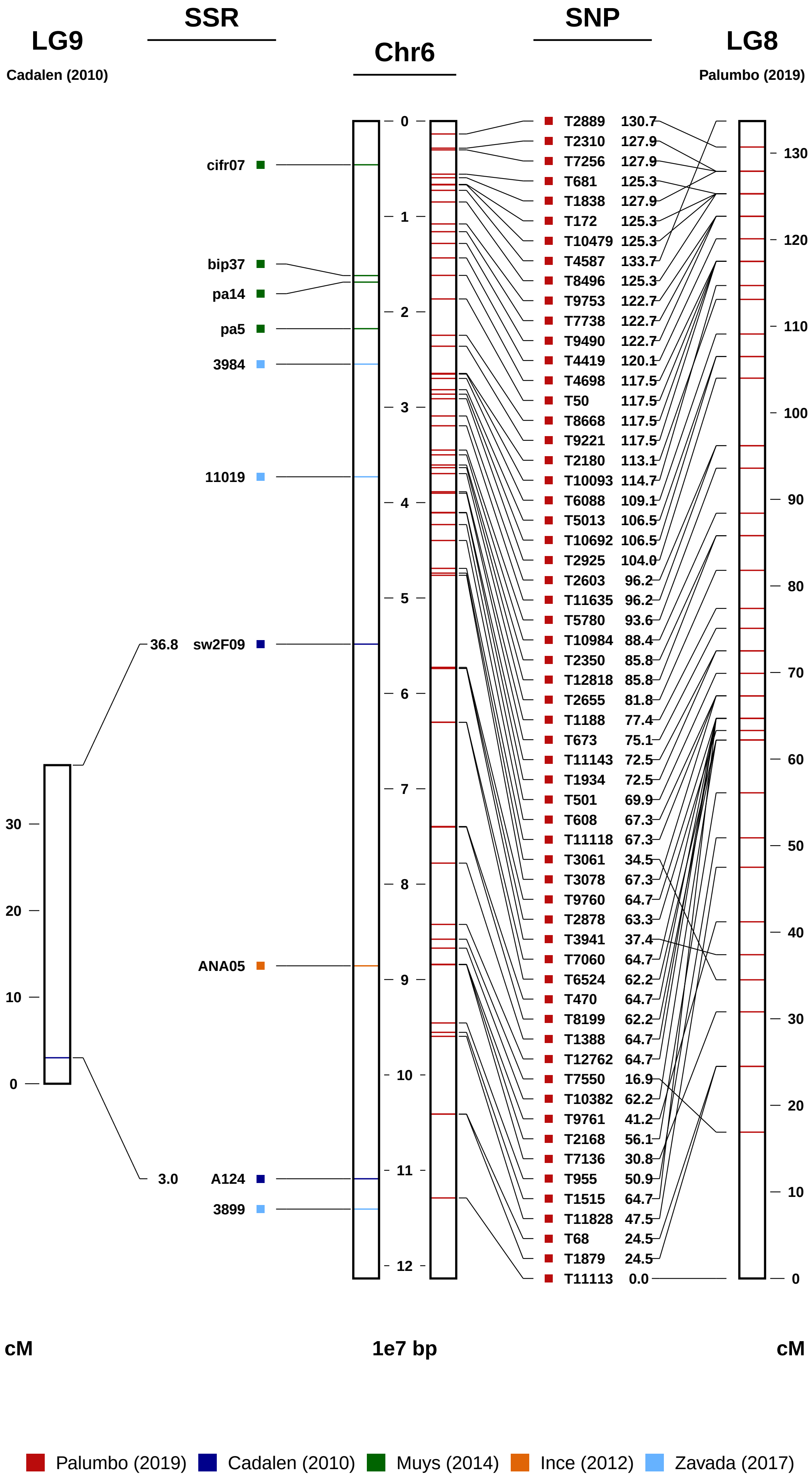

LG9
Cadalen (2010)
SSR
Chr6
SNP
LG8
Palumbo (2019)
cifr07
bip37
pa14
pa5
3984
11019
36.8 sw2F09
ANA05
3.0 A124
3899
0 1 2 3 4 5 6 7 8 9 10 11 12
30 20 10 0
T2889 130.7
T2310 127.9
T7256 127.9
T681 125.3
T1838 127.9
T172 125.3
T10479 125.3
T4587 133.7
T8496 125.3
T9753 122.7
T7738 122.7
T9490 122.7
T4419 120.1
T4698 117.5
T50 117.5
T8668 117.5
T9221 117.5
T2180 113.1
T10093 114.7
T6088 109.1
T5013 106.5
T10692 106.5
T2925 104.0
T2603 96.2
T11635 96.2
T5780 93.6
T10984 88.4
T2350 85.8
T12818 85.8
T2655 81.8
T1188 77.4
T673 75.1
T11143 72.5
T1934 72.5
T501 69.9
T608 67.3
T11118 67.3
T3061 34.5
T3078 67.3
T9760 64.7
T2878 63.3
T3941 37.4
T7060 64.7
T6524 62.2
T470 64.7
T8199 62.2
T1388 64.7
T12762 64.7
T7550 16.9
T10382 62.2
T9761 41.2
T2168 56.1
T7136 30.8
T955 50.9
T1515 64.7
T11828 47.5
T68 24.5
T1879 24.5
T11113 0.0
130 120 110 100 90 80 70 60 50 40 30 20 10 0
cM
1e7 bp
cM
Palumbo (2019) Cadalen (2010) Muys (2014) Ince (2012) Zavada (2017)

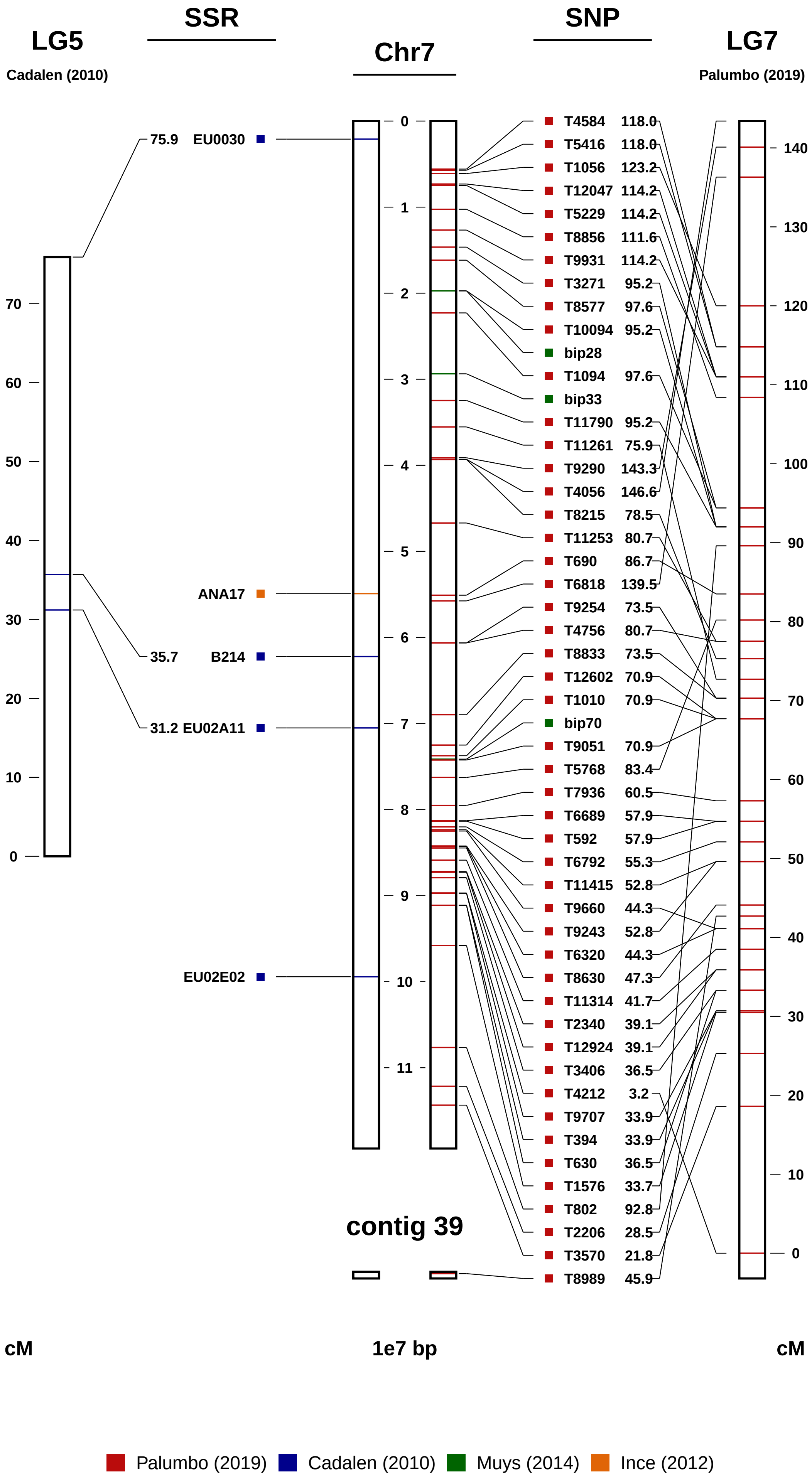
LG5
Cadalen (2010)
SSR
Chr7
SNP
LG7
Palumbo (2019)
75.9 EU0030
ANA17
35.7 B214
31.2 EU02A11
EU02E02
T4584 118.0
T5416 118.0
T1056 123.2
T12047 114.2
T5229 114.2
T8856 111.6
T9931 114.2
T3271 95.2
T8577 97.6
T10094 95.2
bip28
T1094 97.6
bip33
T11790 95.2
T11261 75.9
T9290 143.3
T4056 146.6
T8215 78.5
T11253 80.7
T690 86.7
T6818 139.5
T9254 73.5
T4756 80.7
T8833 73.5
T12602 70.9
T1010 70.9
bip70
T9051 70.9
T5768 83.4
T7936 60.5
T6689 57.9
T592 57.9
T6792 55.3
T11415 52.8
T9660 44.3
T9243 52.8
T6320 44.3
T8630 47.3
T11314 41.7
T2340 39.1
T12924 39.1
T3406 36.5
T4212 3.2
T9707 33.9
T394 33.9
T630 36.5
T1576 33.7
T802 92.8
T2206 28.5
T3570 21.8
T8989 45.9
contig 39
cM
1e7 bp
cM
Palumbo (2019)
Cadalen (2010)
Muys (2014)
Ince (2012)

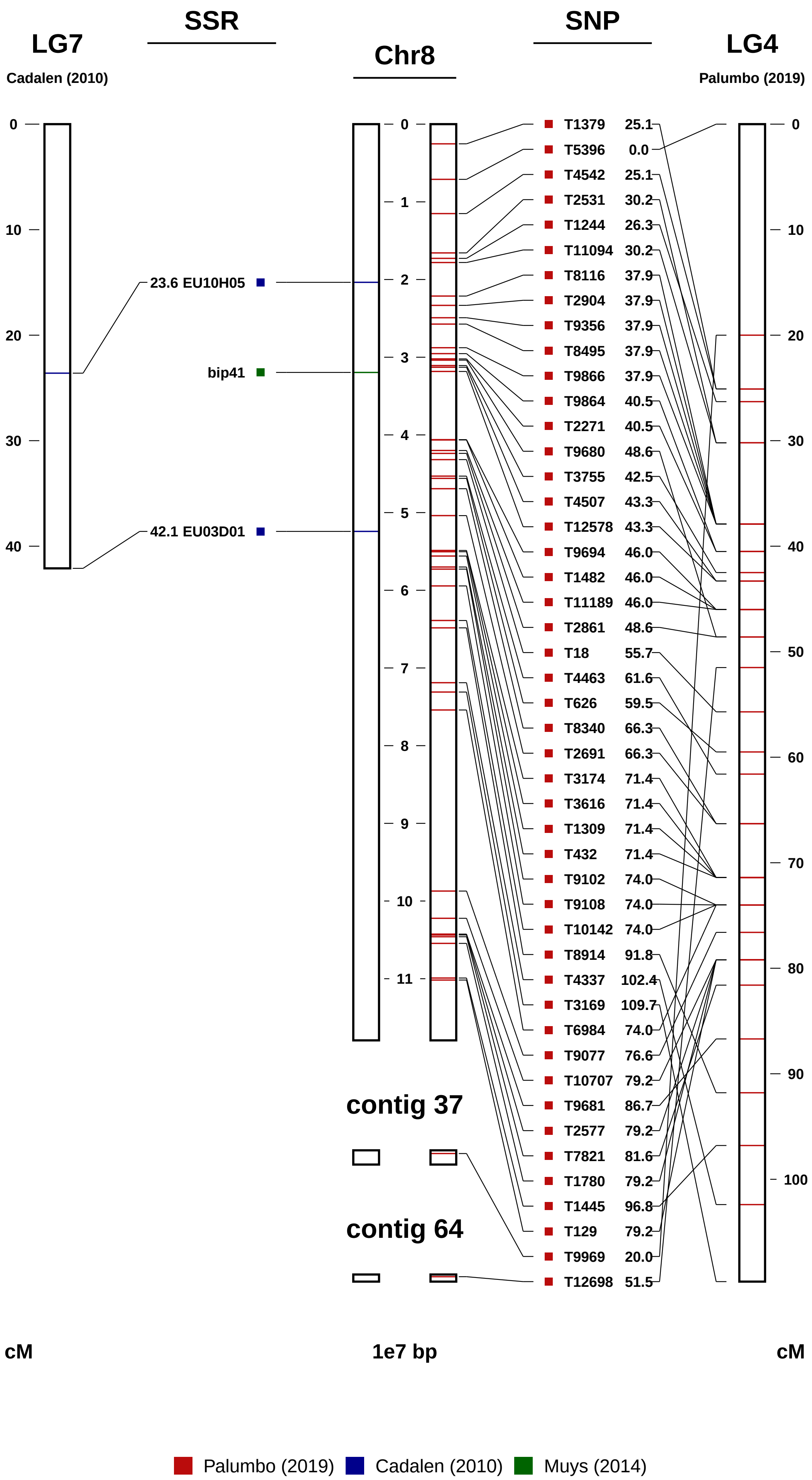

LG7
Cadalen (2010)
SSR
Chr8
SNP
LG4
Palumbo (2019)
23.6 EU10H05
bip41
42.1 EU03D01
T1379 25.1
T5396 0.0
T4542 25.1
T2531 30.2
T1244 26.3
T11094 30.2
T8116 37.9
T2904 37.9
T9356 37.9
T8495 37.9
T9866 37.9
T9864 40.5
T2271 40.5
T9680 48.6
T3755 42.5
T4507 43.3
T12578 43.3
T9694 46.0
T1482 46.0
T11189 46.0
T2861 48.6
T18 55.7
T4463 61.6
T626 59.5
T8340 66.3
T2691 66.3
T3174 71.4
T3616 71.4
T1309 71.4
T432 71.4
T9102 74.0
T9108 74.0
T10142 74.0
T8914 91.8
T4337 102.4
T3169 109.7
T6984 74.0
T9077 76.6
T10707 79.2
T9681 86.7
T2577 79.2
T7821 81.6
T1780 79.2
T1445 96.8
T129 79.2
T9969 20.0
T12698 51.5
contig 37
contig 64
cM
1e7 bp
cM
Palumbo (2019)
Cadalen (2010)
Muys (2014)

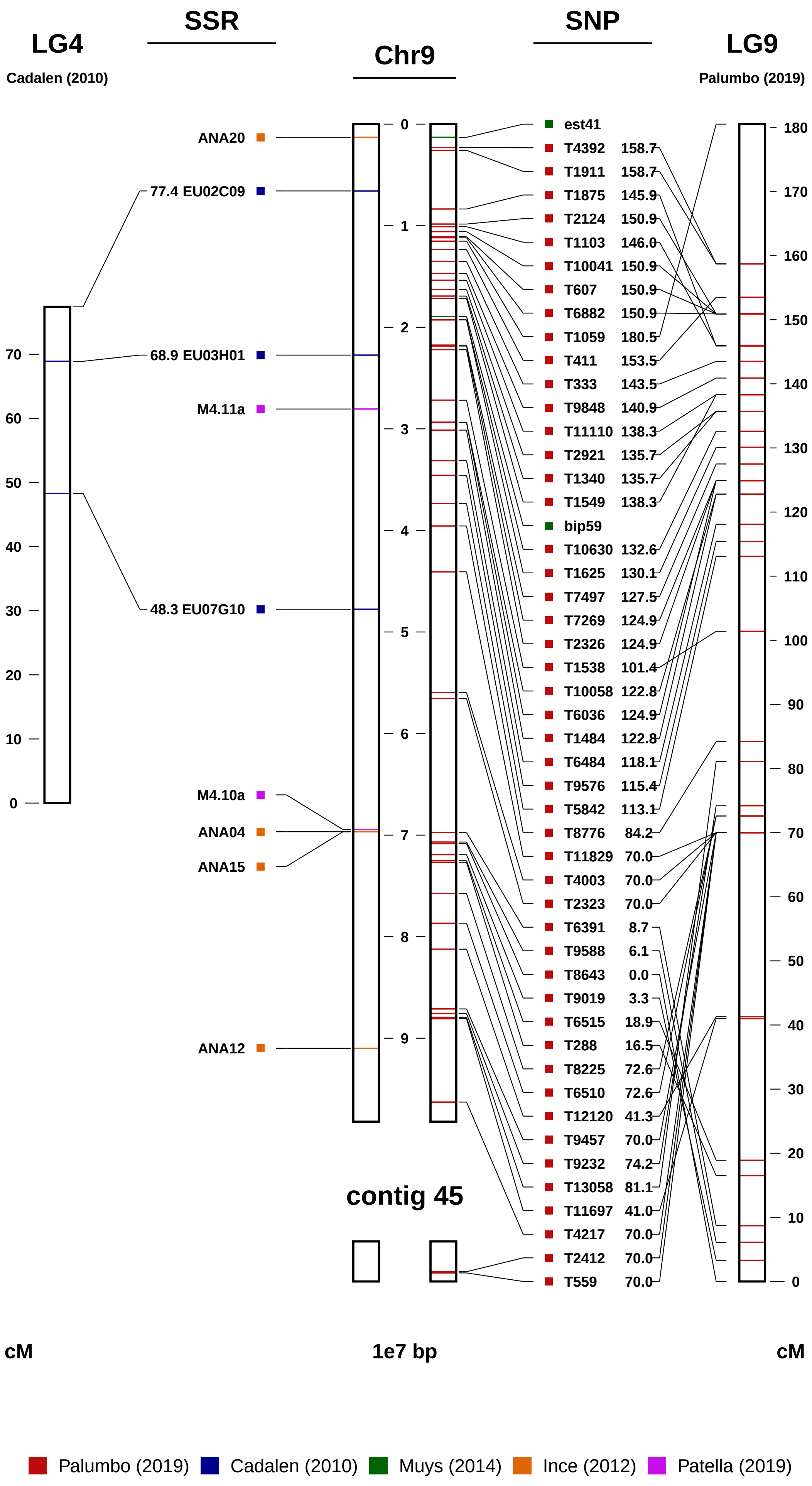

LG4
Cadalen (2010)
SSR
Chr9
SNP
LG9
Palumbo (2019)
ANA20
77.4 EU02C09
68.9 EU03H01
M4.11a
48.3 EU07G10
M4.10a
ANA04
ANA15
ANA12
contig 45
est41
T4392 158.7
T1911 158.7
T1875 145.9
T2124 150.9
T1103 146.0
T10041 150.9
T607 150.9
T6882 150.9
T1059 180.5
T411 153.5
T333 143.5
T9848 140.9
T11110 138.3
T2921 135.7
T1340 135.7
T1549 138.3
bip59
T10630 132.6
T1625 130.1
T7497 127.5
T7269 124.9
T2326 124.9
T1538 101.4
T10058 122.8
T6036 124.9
T1484 122.8
T6484 118.1
T9576 115.4
T5842 113.1
T8776 84.2
T11829 70.0
T4003 70.0
T2323 70.0
T6391 8.7
T9588 6.1
T8643 0.0
T9019 3.3
T6515 18.9
T288 16.5
T8225 72.6
T6510 72.6
T12120 41.3
T9457 70.0
T9232 74.2
T13058 81.1
T11697 41.0
T4217 70.0
T2412 70.0
T559 70.0
cM
1e7 bp
cM
Palumbo (2019)
Cadalen (2010)
Muys (2014)
Ince (2012)
Patella (2019)
