# Supplementary material for: Genome-Wide Datasets of Chicories (Cichorium intybus L.) for Marker-Assisted Crop Breeding Applications: A Systematic Review and Meta-Analysis
Source: Int J Mol Sci. 2023 Jul 19;24(14):11663. doi: 10.3390/ijms241411663 (PMC10380310; doi:10.3390/ijms241411663)
Supplement: Supplementary file 1 [file ijms-24-11663-s001.zip › Figure S2.pdf]

Figure S2: Reference Marker Assisted Selection (MAS) map in chicory. On the left are the genes with their trait-identity number (in the form of apex; 1: Reproductive barriers; 2: Sesquiterpene Lactone biosynthesis (STL); 3: Hydroxycinnamates biosynthesis (HCAs); 4: Inulin metabolism; 5: Stress response; 6: Blue-lilac color; 7: Flowering time; 8: Somatic embryogenesis; 9: Red discoloration; 10: Gene normalization). Each gene is also described in detail in Table 3 whereas Table S1 reports their respective GenBank accession numbers. On the right are reported the two markers (SSRs or SNPs) closest to each gene. The color of square near each marker refers to the source study of the marker (Cadalen *et al.* [25], Muys *et al.* [27], Palumbo *et al.* [28], Patella *et al.* [13], Ince *et al.* [29], Zavada *et al.* [30])

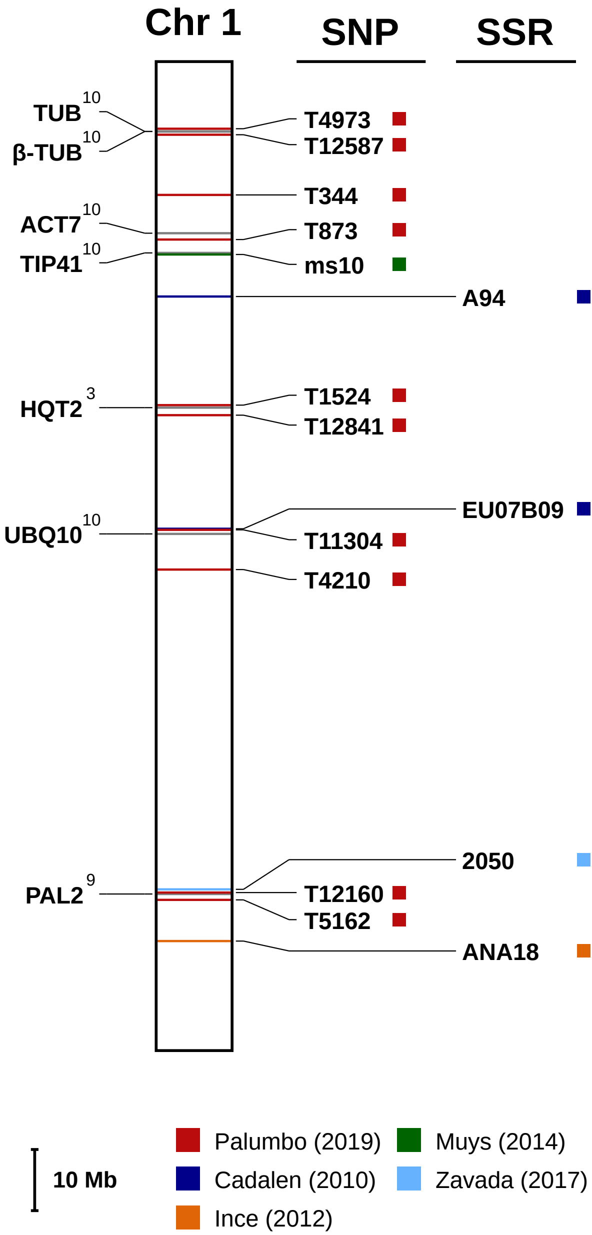
Chr 1
SNP
SSR
TUB 10
β-TUB 10
ACT7 10
TIP41 10
HQT2 3
UBQ10 10
PAL2 9
T4973
T12587
T344
T873
ms10
A94
T1524
T12841
EU07B09
T11304
T4210
2050
T12160
T5162
ANA18
10 Mb
Palumbo (2019)
Cadalen (2010)
Ince (2012)
Muys (2014)
Zavada (2017)

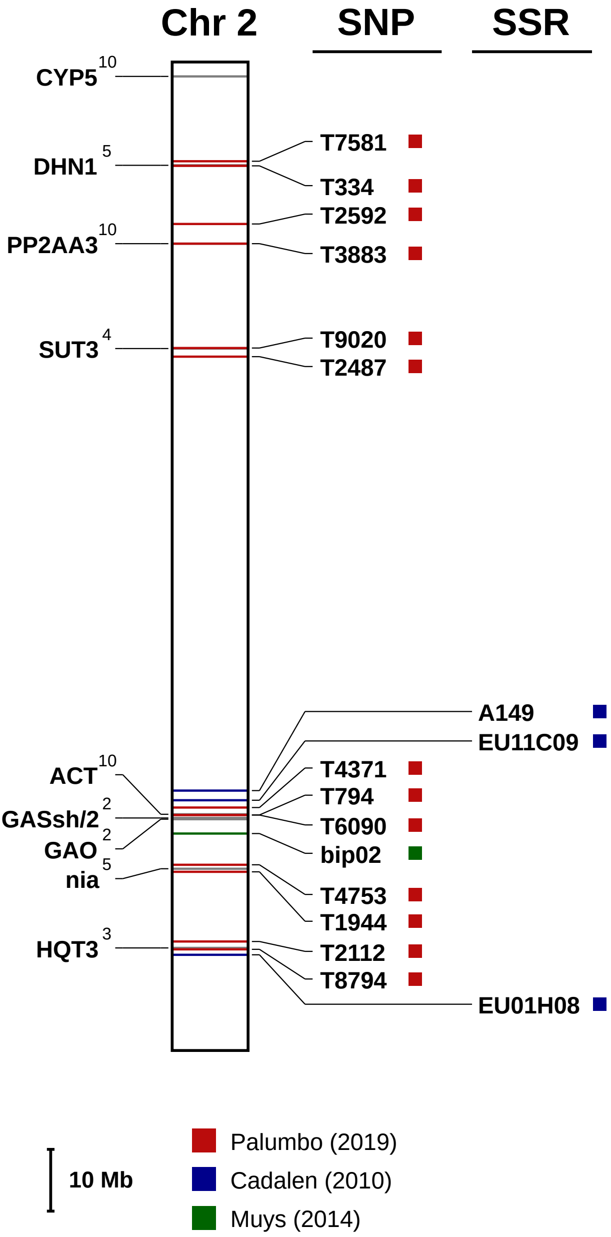
Chr 2
SNP
SSR
CYP5 10
DHN1 5
PP2AA3 10
SUT3 4
ACT 10
GASsh/2 2
GAO 2
nia 5
HQT3 3
T7581
T334
T2592
T3883
T9020
T2487
A149
EU11C09
T4371
T794
T6090
bip02
T4753
T1944
T2112
T8794
EU01H08
10 Mb
Palumbo (2019)
Cadalen (2010)
Muys (2014)

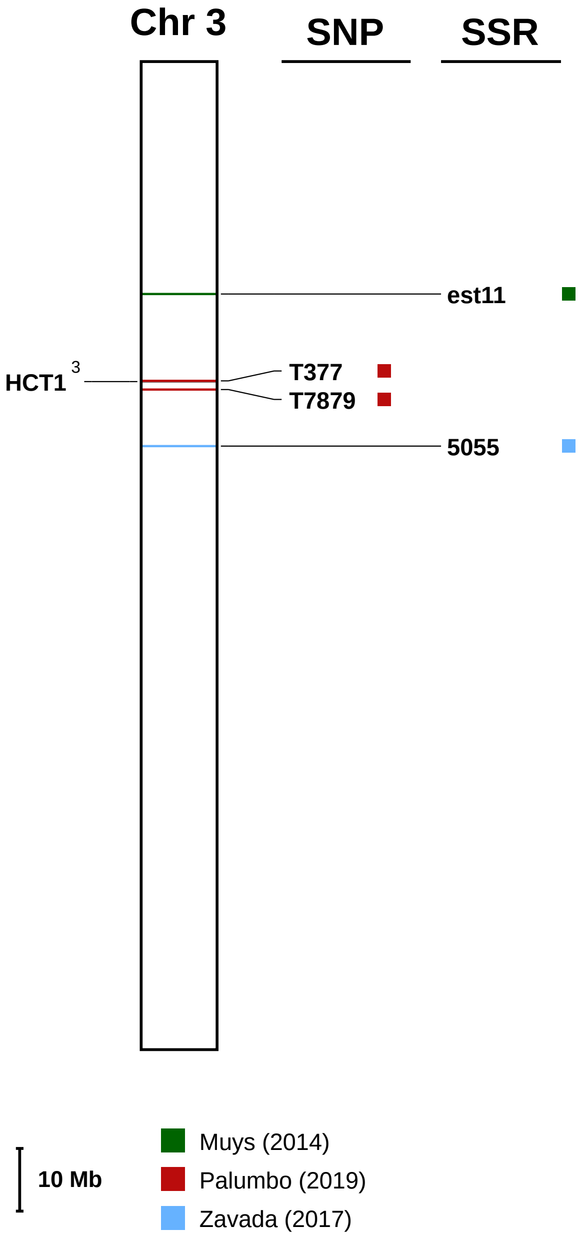

Chr 3
SNP
SSR
est11
HCT1 [3]
T377
T7879
5055
10 Mb
Muys (2014)
Palumbo (2019)
Zavada (2017)

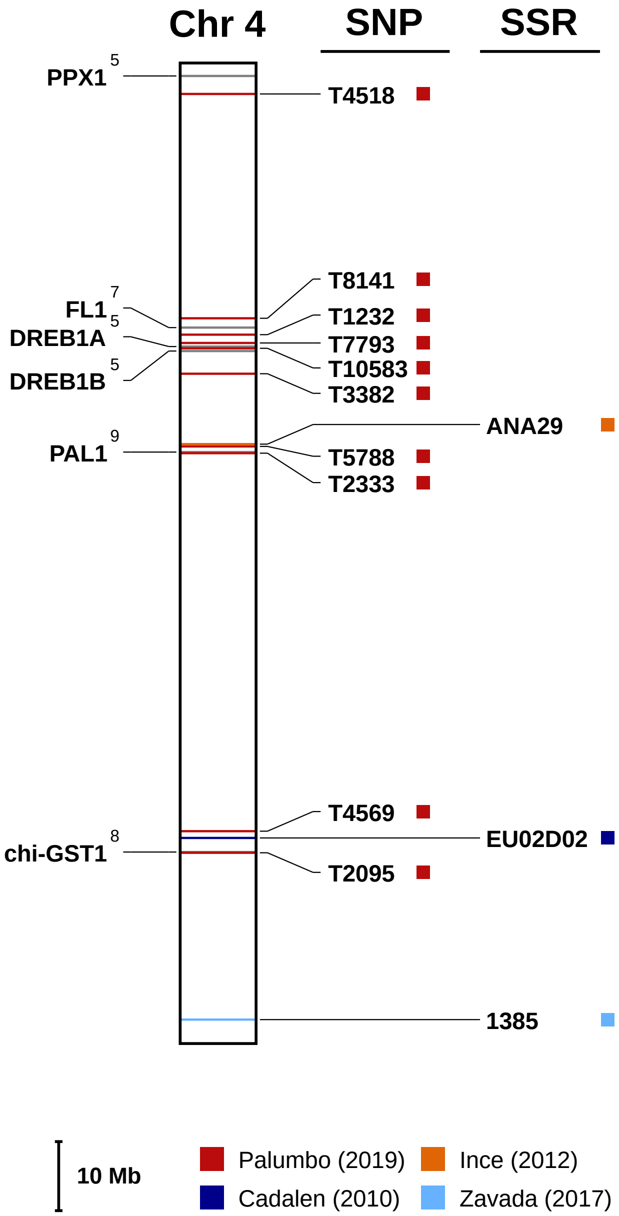

Chr 4
SNP
SSR
PPX1 5
T4518
T8141
FL1 7
T1232
DREB1A 5
T7793
T10583
DREB1B 5
T3382
ANA29
PAL1 9
T5788
T2333
T4569
EU02D02
chi-GST1 8
T2095
1385
10 Mb
Palumbo (2019)
Ince (2012)
Cadalen (2010)
Zavada (2017)

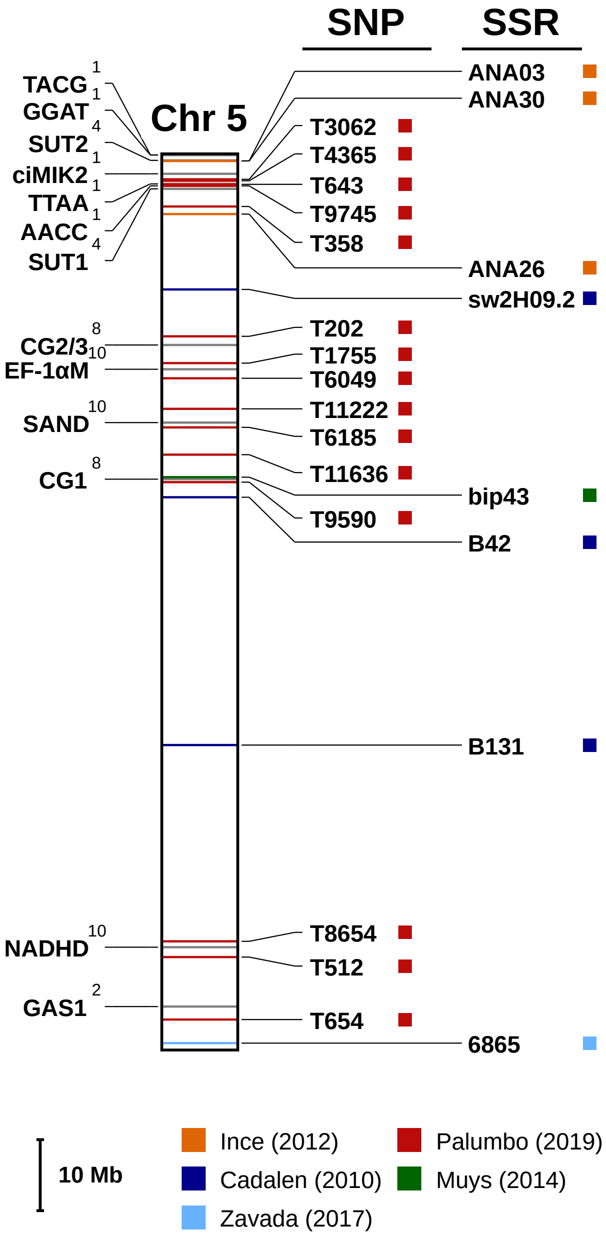

Chr 5
SNP
SSR
TACG 1
GGAT 1
SUT2 4
ciMIK2 1
TTAA 1
AACC 1
SUT1 4
CG2/3 8
EF-1αM 10
SAND 10
CG1 8
NADHD 10
GAS1 2
ANA03
ANA30
T3062
T4365
T643
T9745
T358
ANA26
sw2H09.2
T202
T1755
T6049
T11222
T6185
T11636
bip43
T9590
B42
B131
T8654
T512
T654
6865
10 Mb
Ince (2012)
Palumbo (2019)
Cadalen (2010)
Muys (2014)
Zavada (2017)

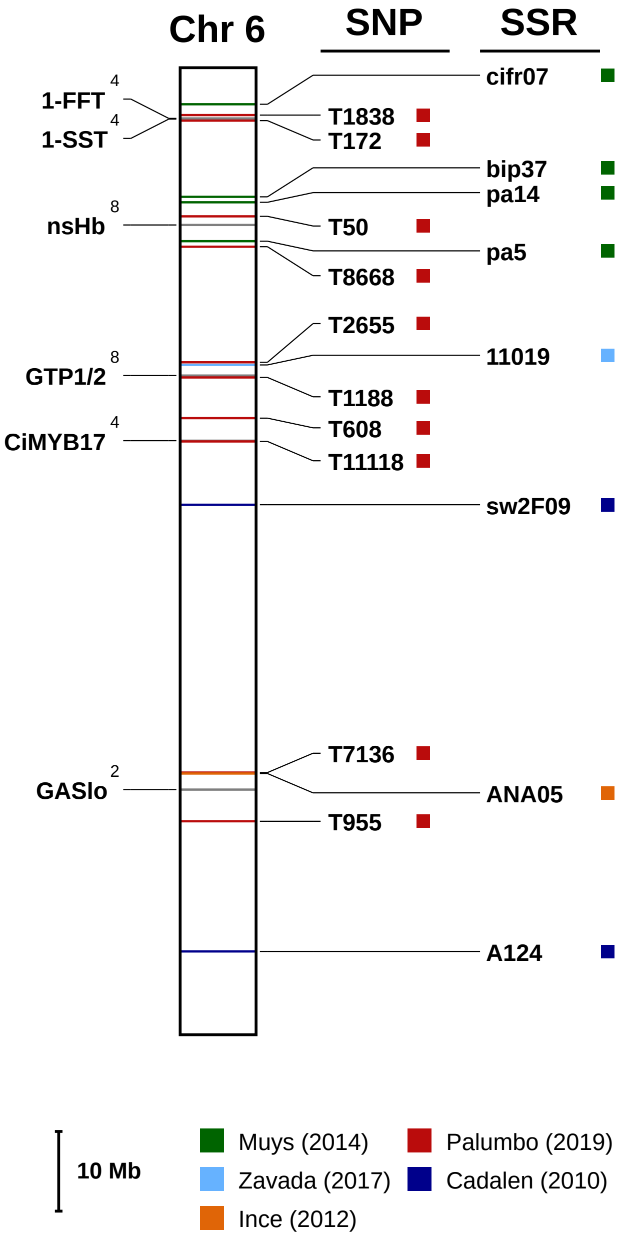

Chr 6
SNP
SSR
4
1-FFT
4
1-SST
8
nsHb
8
GTP1/2
4
CiMYB17
2
GASlo
cifr07
T1838
T172
bip37
pa14
T50
pa5
T8668
T2655
11019
T1188
T608
T11118
sw2F09
T7136
ANA05
T955
A124
10 Mb
Muys (2014)
Palumbo (2019)
Zavada (2017)
Cadalen (2010)
Ince (2012)

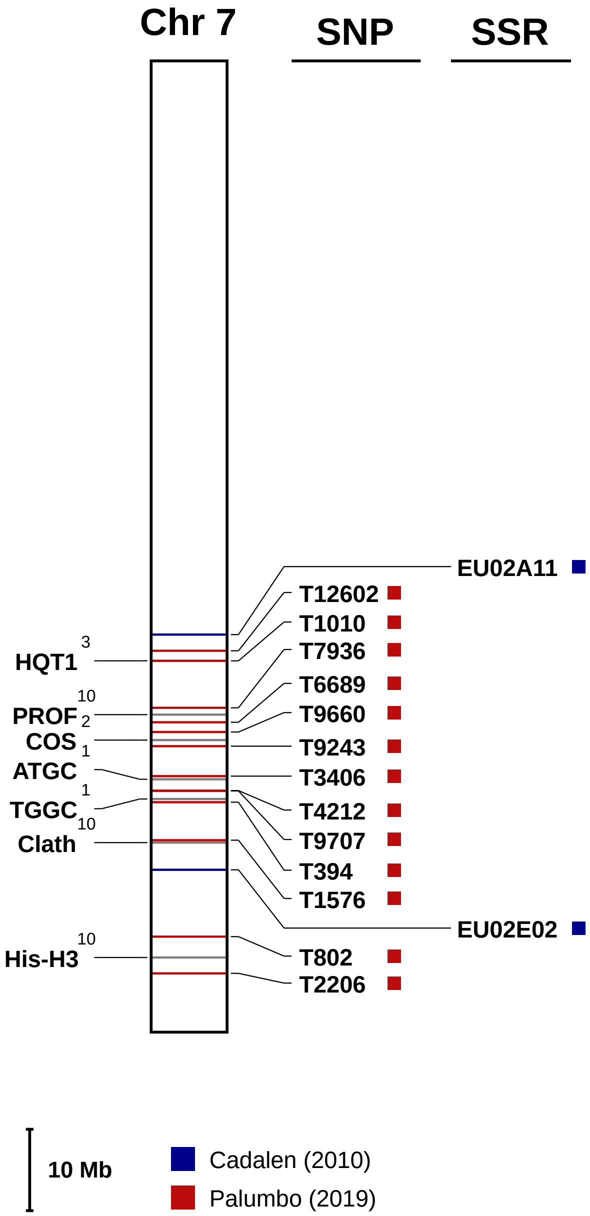

Chr 7
SNP
SSR
EU02A11
T12602
T1010
T7936
T6689
T9660
T9243
T3406
T4212
T9707
T394
T1576
EU02E02
T802
T2206
3
HQT1
10
PROF
2
COS
1
ATGC
1
TGGC
10
Clath
10
His-H3
10 Mb
Cadalen (2010)
Palumbo (2019)

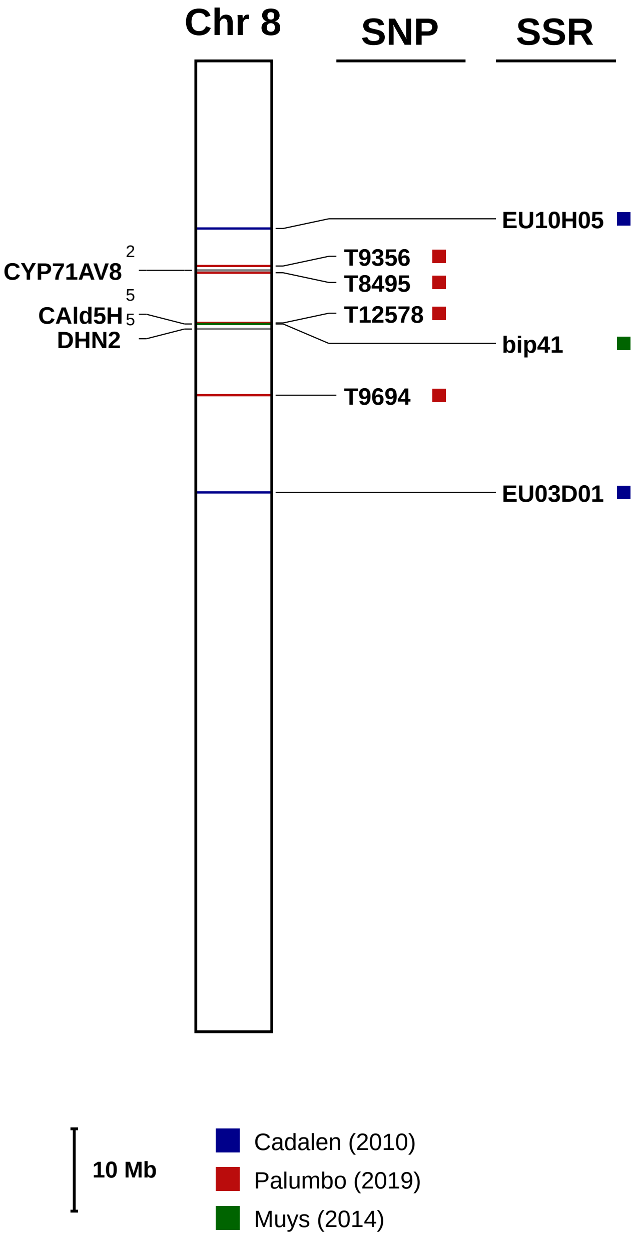
Chr 8
SNP
SSR
EU10H05
T9356
CYP71AV8
2
T8495
5
T12578
CAld5H
5
DHN2
bip41
T9694
EU03D01
10 Mb
Cadalen (2010)
Palumbo (2019)
Muys (2014)

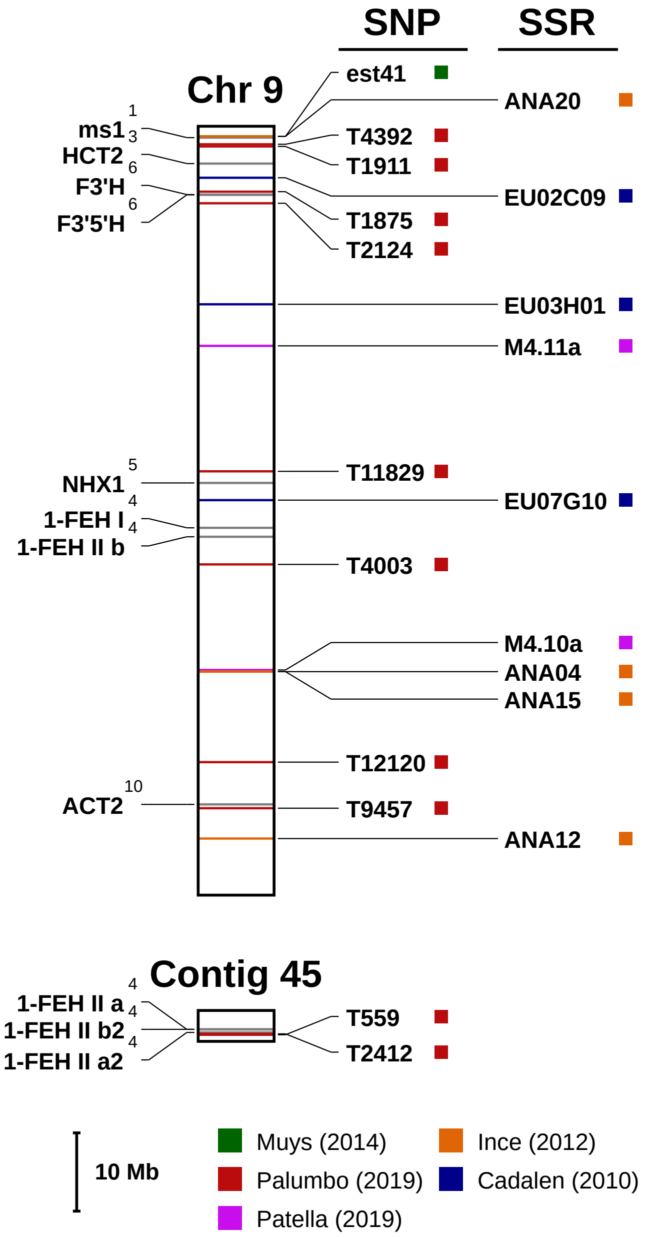

SNP
SSR
Chr 9
est41
ANA20
T4392
T1911
EU02C09
T1875
T2124
ms1
HCT2
F3'H
F3'5'H
1
3
6
6
EU03H01
M4.11a
T11829
EU07G10
NHX1
1-FEH I
1-FEH II b
5
4
4
T4003
M4.10a
ANA04
ANA15
T12120
T9457
ANA12
ACT2
10
Contig 45
1-FEH II a
1-FEH II b2
1-FEH II a2
4
4
4
T559
T2412
10 Mb
Muys (2014)
Palumbo (2019)
Patella (2019)
Ince (2012)
Cadalen (2010)
